# Beetroot Juice and Exercise for Clinical Health and Athletic Performance: A Narrative Review

**DOI:** 10.3390/nu18010151

**Published:** 2026-01-01

**Authors:** Eunjoo Lee, Hun-Young Park, Yerin Sun, Jae-Ho Choi, Seungyeon Woo, Sohyang Cho, Suyoung Kim, Yuanning Zheng, Sung-Woo Kim, Kiwon Lim

**Affiliations:** 1 Physical Activity and Performance Institute, Konkuk University, Seoul 05029, Republic of Korea; eunjooo@konkuk.ac.kr (E.L.); parkhy1980@konkuk.ac.kr (H.-Y.P.); kswrha@konkuk.ac.kr (S.-W.K.); 2 Department of Sports Medicine and Science, Graduate School, Konkuk University, Seoul 05029, Republic of Korea; edre82@konkuk.ac.kr (Y.S.); zas1135@konkuk.ac.kr (J.-H.C.); wsyzz92@konkuk.ac.kr (S.W.); whthgiddl123@konkuk.ac.kr (S.C.); syk6919@konkuk.ac.kr (S.K.); qw1204153841@konkuk.ac.kr (Y.Z.); 3 Department of Physical Education, Konkuk University, Seoul 05029, Republic of Korea

**Keywords:** beetroot juice, dietary nitrate, nitric oxide, vascular function, metabolic health, neuromuscular function, cognitive performance, exercise performance

## Abstract

Beetroot juice (BRJ), a concentrated dietary source of nitrate alongside betalains and polyphenols, influences physiology through enhanced nitrate–nitrite–NO bioavailability, antioxidant activity, and interactions with oral and gut nitrate-reducing microbiota. The efficiency of these mechanisms depends on dose, timing, and preservation of oral bacteria, with antibacterial mouthwash or thiocyanate-rich foods potentially blunting NO_2_^−^ generation. Acute BRJ ingestion consistently elevates circulating nitrate and nitrite, yet its impact on glucose, insulin, and lipid regulation is modest; chronic intake may reinforce nitrate-reduction capacity, improve redox balance, and shift microbial composition, though long-term metabolic outcomes remain variable. Cardiovascular adaptations appear more coherent, with acute reductions in systolic blood pressure and improved endothelial function complemented in some cases by microvascular enhancements during multi-week supplementation. Neuromuscular and cognitive effects are less uniform; BRJ does not reliably increase maximal strength or global cognition but may support electrophysiological recovery after muscle-damaging exercise and improve executive performance under fatigue. In exercise settings, dose and timing are critical, as BRJ most consistently benefits endurance performance by reducing oxygen cost, improving exercise economy, and enhancing time-trial or time-to-exhaustion outcomes, whereas effects on sprint, power, and team-sport tasks are more sensitive to contraction duration, recovery intervals, and athlete training status. Overall, available evidence supports a role for NO-mediated vascular and metabolic pathways in the physiological effects of BRJ, although marked inter-individual variability highlights the need for responder-focused dosing strategies and further mechanistic investigation integrating metabolic, microbial, and performance-related outcomes.

## 1. Introduction

Beetroot juice (BRJ) has attracted growing interest across health and performance research due to its substantial content of inorganic nitrate (NO_3_^−^) along with betalains, polyphenols, and other bioactive compounds [1,2]. The reduction of dietary NO_3_^−^ to nitrite and nitric oxide (NO) through the nitrate–nitrite–NO pathway is now well established, and this mechanism enables NO generation even under low-oxygen or acidic conditions typical of exercise [3]. Because NO influences vascular tone, oxidative balance, mitochondrial efficiency, and muscle contractile behavior, BRJ has become a promising nutritional strategy in several physiological domains [4,5,6]. Initial clinical research highlighted BRJ’s potential through observations of blood pressure reductions and improvements in endothelial function [7,8]. Subsequent studies broadened this perspective by reporting effects on mitochondrial efficiency [9], muscle performance characteristics [10], and changes in cerebral blood flow and cognitive responses [11]. In exercise science, BRJ has been explored for its ability to reduce the oxygen cost of submaximal work, delay fatigue in high-intensity tasks, and modify recovery dynamics [12,13]. At the same time, findings have not been uniform; the magnitude of response depends on exercise modality, supplementation dose and duration, and the individual’s training background [14]. More recently, researchers have emphasized that BRJ’s effects cannot be explained solely by nitrate intake. The timing of supplementation, the integrity of the oral microbiota, dietary habits, and factors such as age or cardiometabolic status all shape the degree to which NO_3_^−^ is converted to NO_2_^−^ and ultimately to NO [8,15]. These interactions underscore the need to interpret BRJ research within a broader physiological and behavioral context.

Therefore, the aim of this narrative review is to synthesize current evidence on the physiological actions of BRJ and to comprehensively examine human studies spanning metabolic, cardiovascular, neuromuscular, cognitive, and exercise-performance outcomes. By considering findings from both acute and chronic supplementation trials, this review explores how dosing strategies, timing, individual characteristics, and contextual factors shape the physiological and functional responses to BRJ. Through this broad evaluation, we summarize the potential applications, practical considerations, and known limitations of BRJ supplementation within both health-related and exercise settings.

This narrative review was designed to provide a broad overview of human evidence on the physiological and functional effects of beetroot juice across health- and exercise-related domains. Relevant literature was identified through searches of PubMed, Scopus, and Web of Science, including English-language studies available up to November 2025, using combinations of keywords related to beetroot juice, dietary nitrate, exercise performance, metabolic health, cardiovascular function, neuromuscular outcomes, immune responses, and cognitive function. Studies investigating the ingestion of isolated dietary nitrate or nitrate salts without beetroot juice were excluded. With the exception of the mechanistic section, which incorporates supporting in vivo and in vitro evidence, this narrative review primarily focuses on human intervention studies directly examining the effects of beetroot juice consumption. Meta-analyses and systematic reviews are cited only in the main text to provide contextual background and are not included in the tables, which summarize individual human intervention trials. As a narrative review, this work does not aim to systematically integrate or quantitatively synthesize findings; therefore, formal inclusion/exclusion criteria and risk-of-bias assessments were not applied. Instead, the review emphasizes broad coverage and descriptive synthesis of relevant studies across the topic.

## 2. Physiological Mechanisms Underlying the Effects of Beetroot Juice

NO is known to be produced endogenously through the oxidation of L-arginine catalyzed by nitric oxide synthase (NOS) [16,17,18]. This oxygen-dependent enzymatic pathway involves three major NOS isoforms—neuronal NOS, inducible NOS, and endothelial NOS —each contributing to essential physiological functions such as vascular tone regulation, glucose uptake, and skeletal muscle blood flow [3,17]. However, recent evidence has identified an alternative, oxygen-independent nitrate–nitrite–NO pathway activated by dietary nitrate (NO_3_^−^), such as that obtained from BRJ [1,2]. Following ingestion, NO_3_^−^ is absorbed in the small intestine and enters systemic circulation, where approximately 20–25% undergoes entero-salivary recirculation and is concentrated in the salivary glands. In the oral cavity, anaerobic bacteria (e.g., *Neisseria*, *Veillonella*) reduce NO_3_^−^ to nitrite (NO_2_^−^) [19]. The resulting NO_2_^−^ is further reduced to NO through non-enzymatic reactions in the highly acidic environment of the stomach (pH 1–3), where nitrous acid decomposes to NO and other reactive nitrogen species [2]. A portion of NO_2_^−^ enters systemic circulation and is enzymatically reduced to NO by deoxygenated hemoglobin (deoxy-Hb), deoxygenated myoglobin (deoxy-Mb), xanthine oxidoreductase (XOR), and mitochondrial respiratory chain enzymes—processes that are further enhanced under hypoxic or acidic conditions [20]. Thus, NO production can be maintained even when NOS activity is impaired.

Once produced, NO diffuses into vascular smooth muscle cells (SMCs) and activates soluble guanylate cyclase (sGC), leading to increased cyclic guanosine monophosphate (cGMP) concentrations, decreased intracellular Ca^2+^, and smooth muscle relaxation, ultimately inducing vasodilation [21,22]. Activation of this NO–sGC–cGMP signaling pathway supports endothelial function, enhances vascular compliance, lowers blood pressure, and promotes capillary recruitment and microvascular perfusion, thereby augmenting oxygen delivery and muscle oxygenation at the tissue level [23,24].

NO also exerts antiplatelet, antithrombotic, and endothelial-protective effects [8,25]. Furthermore, by inhibiting nicotinamide adenine dinucleotide phosphate oxidase and the nuclear factor-kappa B (NF-κB) signaling pathway, NO reduces oxidative stress and inflammatory responses, contributing to an overall improvement in cardiovascular function [4,26,27]. Beyond nitrate, BRJ contains several bioactive compounds—including betalains, betaine, and polyphenols—that provide complementary antioxidant and anti-inflammatory mechanisms [5]. Betalains (e.g., betanin, vulgaxanthin) act as potent antioxidants by directly scavenging reactive oxygen species (ROS), and by activating nuclear factor erythroid 2–related factor 2 (Nrf2). Through the antioxidant response element ARE, Nrf2 upregulates Phase II detoxification enzymes and endogenous antioxidant enzymes, thus attenuating oxidative stress [28,29,30,31]. Betalains and polyphenols further suppress inflammatory mediators—including NF-κB, cyclooxygenase-2, tumor necrosis factor-alpha, and interleukin-6 (IL-6)—thereby reinforcing endothelial protection [5,32].

**Figure 1:**
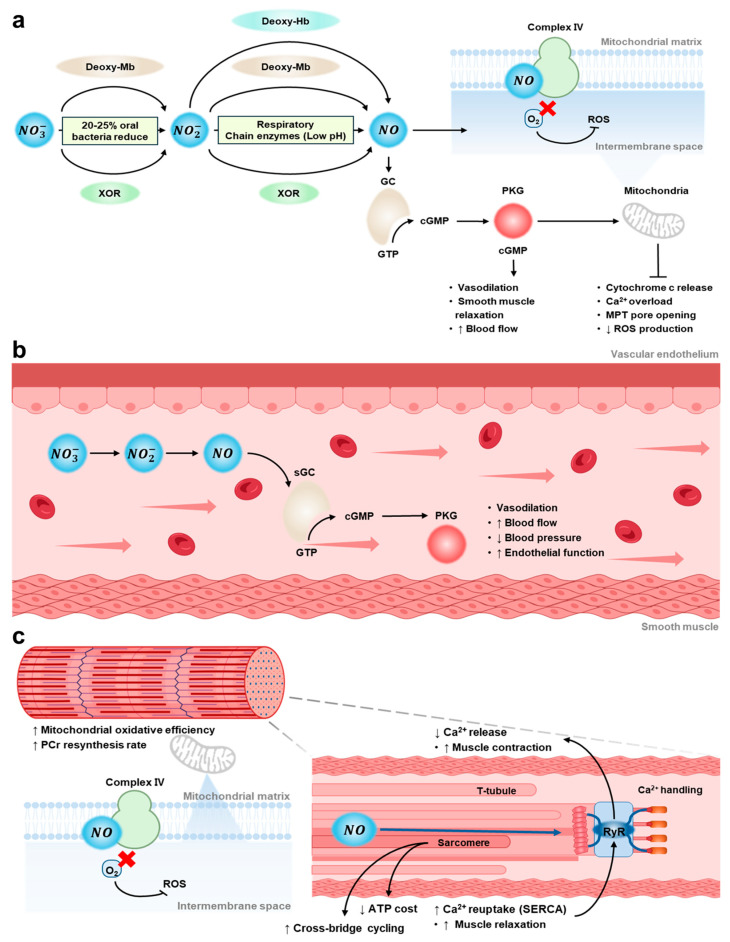
Overview of the physiological mechanisms through which beetroot juice (BRJ) influences nitric oxide availability, vascular function, and skeletal muscle energetics. (**a**) Entero-salivary nitrate reduction and hypoxic/acidic conversion of NO_3_^−^ → NO_2_^−^ → NO, involving deoxygenated Hb/Mb, XOR, and mitochondrial pathways. (**b**) Endothelial NO signaling through the sGC–cGMP–PKG pathway promotes vasodilation, blood flow regulation, and improved vascular function. (**c**) NO effects on skeletal muscle energetics, including enhanced mitochondrial efficiency, faster PCr resynthesis, lower ATP cost, and modulation of Ca^2+^ handling via RyR and SERCA. ↑ and ↓ indicate increases and decreases, respectively. The red cross denotes NO-mediated inhibition or competition of oxygen binding at cytochrome c oxidase (Complex IV).

Betaine acts as a methyl donor to reduce homocysteine concentrations and provides additional anti-inflammatory support to maintain cardiovascular stability [33]. At the skeletal muscle level, NO–cGMP signaling activates protein kinase G (PKG), which modulates intracellular Ca^2+^ homeostasis, enhances sarcoplasmic reticulum Ca^2+^ reuptake, and increases mitochondrial oxidative efficiency [10,34]. These adaptations improve contraction efficiency, reduce oxygen utilization, lower whole-body oxygen consumption (VO_2_), and enhance fatigue resistance [9,35]. Notably, NO competes with oxygen at cytochrome c oxidase (Complex IV), reducing the oxygen cost of ATP production and improving energy efficiency [9,36]. Understanding these physiological pathways (Figure 1) underscores the importance of factors such as nitrate dose, timing of ingestion, population characteristics, and oral microbiome integrity, all of which critically shape the magnitude of BRJ’s effects and are examined in the following section.

## 3. Practical Considerations for Beetroot Juice Supplementation

### 3.1. Timing Strategies for Exercise Settings

Most trials reporting benefits in vascular reactivity or exercise performance have aligned BRJ ingestion with the rise in circulating NO_3_^−^ and NO_2_^−^, which typically show their largest increases 2–3 h after intake [1,12]. This window corresponds to a period when NO formation is more strongly supported by the nitrate–nitrite pathway, and it is also when reductions in oxygen cost during submaximal work have most consistently been observed. Some studies have tested longer intervals, particularly in protocols involving prolonged exercise, yet the 2–3 h timing has remained sufficiently robust across different exercise modalities. When BRJ is consumed across several days or weeks, circulating nitrate gradually stabilizes at a higher baseline, and the exact timing becomes less sensitive; however, maintaining daily ingestion appears essential because the effect dissipates within 24–30 h without continued intake [37]. Testing schedules that diverge markedly from these pharmacokinetic patterns often show weaker results, suggesting that timing is an important contributor to variability in the literature.

### 3.2. Dose–Response and Population-Specific Responsiveness

The nitrate content of commercially available BRJ varies widely, but doses approximating 5–9 mmol of NO_3_^−^ have repeatedly produced measurable changes in NO_2_^−^ levels and improvements in hemodynamic or exercise outcomes [38,39]. Larger doses may be necessary for individuals with higher body mass or reduced vascular or metabolic function due to differences in nitrate distribution volume or impaired conversion efficiency. Still, responses do not scale linearly with dose, and several studies suggest diminishing returns when nitrate intake exceeds the capacity of oral bacteria or systemic pathways to reduce it. Training status adds another layer of complexity. Recreationally active individuals tend to show clearer benefits in VO_2_ kinetics, exercise economy, and blood pressure regulation, whereas highly trained endurance athletes often show minimal or inconsistent improvements, likely because their baseline physiological state already reflects high NO availability and efficient mitochondrial function [14,40]. Dietary background also matters; individuals habitually consuming nitrate-rich vegetables may exhibit blunted responses to supplementation because their baseline nitrite pool is already elevated. These population-specific factors help explain why responses to BRJ vary even when dosing protocols are similar.

### 3.3. Considerations Related to Oral Microbiome Preservation

The reduction of NO_3_^−^ to NO_2_^−^ relies almost entirely on oral nitrate-reducing bacteria, making the composition and activity of the oral microbiome one of the most influential determinants of how effectively BRJ intake translates into physiological changes. The suppressive effect of antibacterial mouthwash is well documented; regular use can markedly lower bacterial nitrate reduction and blunt the expected rise in circulating NO_2_^−^, diminishing the improvements in blood pressure and vascular function reported in many supplementation studies [8,15]. Daily oral-hygiene habits, such as brushing immediately before ingestion or using certain toothpaste ingredients, can also transiently affect nitrate metabolism, although the magnitude of these effects varies. Dietary choices add another layer of nuance. Cruciferous vegetables such as broccoli, cauliflower, and red cabbage—along with related varieties like kale, Brussels sprouts, and Bok choy—contain appreciable amounts of thiocyanate, a compound that can compete with nitrate reduction under some conditions. While their influence is generally weaker than that of antimicrobial rinses, frequent or high intake may alter the oral environment enough to shift NO_2_^−^ formation in sensitive individuals. Because these microbial and dietary factors operate at the first step of nitrate metabolism, even small disruptions can propagate downstream and partly explain the heterogeneous responses often seen across BRJ trials. Preserving the typical activity of oral nitrate-reducing bacteria is therefore essential when interpreting findings or advising individuals on how to use BRJ for vascular, metabolic, or performance-related purposes.

## 4. Effects of Beetroot Juice in Health and Exercise

**Figure 2:**
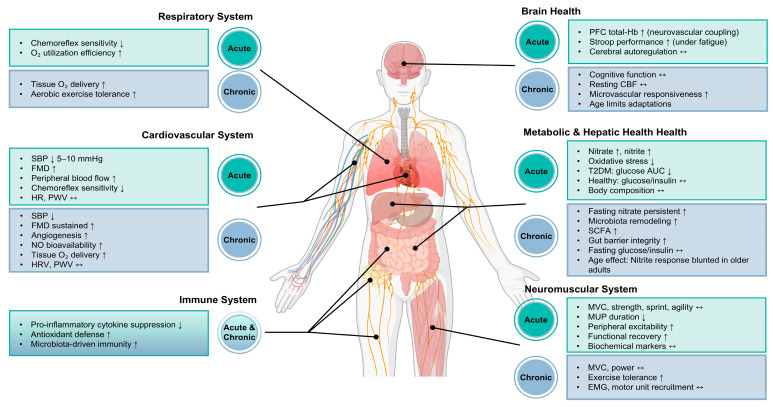
Overview of acute and chronic health-related outcomes of beetroot juice supplementation across physiological systems. ↑ and ↓ indicate increases and decreases, respectively, while ↔ denotes no apparent change.

The physiological actions of BRJ extend across multiple systems, and human studies have examined these effects in metabolic, cardiovascular, neuromuscular, immune, and cognitive domains. Although the magnitude of response varies across individuals and study designs, patterns in the literature suggest that changes in NO availability, microvascular function, mitochondrial efficiency, and redox balance are thought to contribute to several of the reported outcomes. However, the magnitude and consistency of these effects vary substantially across studies, reflecting heterogeneity in participant characteristics, supplementation protocols, and outcome measures. Figure 2 provides an overview of acute and chronic responses across organ systems and illustrates how these physiological pathways align with the findings summarized in this section.

### 4.1. Metabolic Health

Metabolic health encompasses glucose and insulin regulation, lipid profiles, oxidative balance, and contributions from the oral–gut microbiome. BRJ contains inorganic nitrate alongside betalains, polyphenols, and organic acids, allowing multiple routes of metabolic influence. Key findings from acute interventions are summarized in Table 1, while chronic adaptations are presented in Table 2.

Acute BRJ ingestion reliably raises circulating nitrate and nitrite [41,42]. Compared with sodium nitrate, beetroot juice uniquely increases betaine and choline [42], reflecting its broader composition. Despite clear biochemical changes, acute supplementation produces minimal effects on body composition or lipid markers [39,43,44,45,46,47,48]. Glucose-related outcomes remain stable in healthy adults [46], although reductions in total glucose exposure have been reported in type 2 diabetes (T2D) without concurrent changes in insulin [45]. Metabolomics analyses describe characteristic fingerprints—including dopamine-3-O-sulfate and 4-methylpyridine-2-carboxylic acid—that further increase during exercise [49].

Chronic BRJ ingestion yields patterns distinct from acute responses. Long-term intake preserves acute nitrite responsiveness and elevates fasting nitrate [39]. Four-week supplementation increased Neisseria and reduced Veillonella, correlating with higher plasma nitrate/nitrite [50]. Older adults show blunted nitrite responses, with greater ceruloplasmin-related NO scavenging [51]. Gut microbial shifts—including increased Akkermansia, decreased Bacteroides fragilis, and higher short-chain fatty acids (SCFAs) levels—have also been observed [52]. Chronic intake reduces oxidative stress and maintains NO availability even with impaired renal clearance [43]. In contrast, fasting glucose, insulin, homeostatic model assessment-insulin resistance (HOMA-IR), and postprandial responses largely remain unchanged [46,47,48]. These metabolic patterns vary with supplementation duration, dose, and individual microbial profiles, and the specific physiological relevance may differ across populations.

**Table 1:** Acute effects of beetroot juice supplementation on metabolic health outcomes.

Reference	Participants	Experimental Conditions	Supplementation Protocol	Supplement Source	Variables	Results
Fuchs et al. [44]	M, obese, insulin-resistant (*n* = 16)	EC1: BRJ EC2: CON	EC1: BRJ 70 mL/day (4.8 mmol) (180 min before)/1 day	Beet It (James White Drinks Ltd., Ipswich, UK)	Glucose homeostasis, Conduit Artery Blood Flow, Vascular occlusion plethysmography, NIRS, BP	Vascular resistance ↓ Postprandial glucose ↔ Postprandial insulin ↔
Garnacho-Castaño et al. [41]	M, healthy with ≥ 2 years of crossfit experience (*n* = 12)	EC1: BRJ EC2: PLA	EC1: BRJ 140 mL/day (12.8 mmol) (180 min before)/1 day	Beet It (James White Drinks Ltd., Ipswich, UK)	Workout of the day Test (CrossFit), Blood Sampling, SpO_2_, CMJ	(1st) Number of Repetitions ↑ (2nd) Number of Repetitions ↔ Serum Cortisol SpO_2_ ↓ Muscle Fatigue ↓ Serum Testosterone ↔ Testosterone/cortisol Ratio ↔ Blood Lactate ↔
Giampaoli et al. [49]	M (*n* = 2) and W (*n* = 5) healthy adult (*n* = 7)	EC1: BRJ EC2: BRJ + EXE EC3: PLA + EXE	EC1: BRJ 200 mL/day (9.7 mmol) (60 min before)/1 day EC2: BRJ 200 mL/day (9.7 mmol) (60 min before)/1 day EC3: BRJ 20 mL/day (1.8 mmol) (60 min before)/1 day	Commercial BRJ (Aureli Mario SS Agricola, Ortucchio, Italy)	Cycle Ergometer, Urine Sampling, Gas Exchange	Dopamin-3-O-sulfate ↑ 4-methylpyridine-2-carboxylic acid ↔
Heredia-Martínez et al. [43]	M (*n* = 7) and W (*n* = 8) hemodialysis (*n* = 8) and healthy adult (*n* = 15)	EC1: BRJ (HD) EC2: BRJ EC3: PLA (HD) EC4: PLA	EC1: BRJ 70 mL/day (6.4 mmol) (NR)/1 day EC2: BRJ 70 mL/day (6.4 mmol) (NR)/1 day	Beetroot Juice (James White Drinks Ltd., Ipswich, UK)	Blood Sampling, Dialysate Sampling, Saliva Sampling, BP	(EC1 vs. EC2) Plasma NO_2_^−^ & NO_3_^−^ ↑ (EC1 vs. EC2) Plasma NO_2_^−^ AUClast ↑ (EC1) Plasma NO_2_^−^ AUClast & t ½ ↑ (EC1 vs. EC2) BP and Plasma cGMP ↔ (EC1) Potassium and Safety and Tolerability ↔
Jurga et al. [42]	M (*n* = 3) and W (*n* = 5) healthy adult (*n* = 8)	EC1: BRJ EC2: NIT	EC1: BRJ 2 × 70 mL/day (12.9 mmol) (60 min before)/1 day	Beet It Sport (James White Drinks)	Blood Sampling, Flow-mediated skin fluorescence	Plasma NO_x_ ↑ Plasma Betaine/Choline ↑ Trimethylamine/ Trimethylamine *N*-oxide ↔
Rowland et al. [53]	M, healthy adult (*n* = 12)	EC1: BRJ (MORN) EC2: BRJ (AFT) EC3: BRJ (EVE) EC4: PLA (MORN) EC5: PLA (AFT) EC6: PLA (EVE)	EC1: BRJ 2 × 70 mL/day (13 mmol) (morning)/1 day EC2: BRJ 2 × 70 mL/day (13 mmol) (mid-day)/1 day EC3: BRJ 2 × 70 mL/day (13 mmol) (evening)/1 day	Beet It (James White Drinks Ltd., Ipswich, UK)	Cycle Ergometer Severe-Intensity Exercise, TTE, Urine Sampling, Saliva Sampling, BP, PWV	(EC1 & EC2 & EC3) NO_3_^−^ Metabolism ↑ (Time) NO_3_^−^ Metabolism ↔ (EC1 & EC2 & EC3) Central SBP ↓ (Time) SBP ↔ (EC1 & EC2 & EC3 vs. Time) Brachial SBP ↔ (EC1 & EC2 & EC3 vs. Time) TTE ↔
Shepherd et al. [46]	M (*n* = 19) and W (*n* = 12) healthy younger and older (*n* = 31)	EC1: BRJ (YN) EC: BRJ (OLD) EC3: PLA (YN) EC4: PLA (OLD)	EC1: BRJ 140 mL/day (11.9 mmol) (morning)/1 day EC2: BRJ 140 mL/day (11.9 mmol) (morning)/1 day	Beet It (James White Drinks., Ipswich, UK)	Magnetic resonance imaging, Incretin and *C*-peptide, Blood Sampling	(EC1 & EC2) Plasma NO_2_^−^ ↑ (EC1 & EC2) Plasma NO_2_^−^ ↑ (EC1 & EC2) Portal vein flux ↔ (EC1) Portal vein velocity ↓ (EC2) Portal vein velocity ↔ (EC1 & EC2) Plasma glucose, Total GLP-1, Active GLP-1, *C*-peptide, SBP, DBP ↔
Tyler et al. [45]	M (*n* = 2) and W (*n* = 5) T2D adult (*n* = 7)	EC1: BRJ EC2: PLA	EC1: BRJ 70 mL/day (6.4 mmol) (120 min before)/1 day	Beetroot Juice (James White Drinks, Ipswich., UK)	Blood Sampling, OGTT, BP	Glucose AUG ↓ ΔSVR ↓ Salivary NO_2_^−^ & NO_3_^−^ ↑ SBP & DBP ↔ HOMA-IR & QUICKI ↔

Note. Arrows indicate statistically significant differences as reported in the original studies (↑ increase, ↓ decrease); ↔ indicates no statistically significant difference.

**Table 2:** Chronic effects of beetroot juice supplementation on metabolic health outcomes.

Reference	Participants	Experimental Conditions	Supplementation Protocol	Supplement Source	Variables	Results
Alharbi et al. [48]	M (*n* = 7) and W (*n* = 22) obesity mid-age and older (*n* = 29)	EC1: CR + BRJ EC2: CR	EC1: BRJ 70 mL/day (6.4 mmol) (morning)/2 weeks	Beet It (James White Ltd., Ashbocking, Suffolk, UK)	BP, REE, Handgrip Strength, Skin microvascular blood flow, IPAQ, Urine Sampling, Saliva Sampling	Average Microvascular Flux ↑ NO-dependent Endothelial Activity ↑ SBP ↓ Cognitive Function ↑ Oxidative Stress ↑ NO bioavailability ↑ Physical Strength ↑ Metabolic Adaptation ↔ Body composition ↔
Babateen et al. [47]	M (*n* = 24) and W (*n* = 38) overweight/obese older adults (*n* = 62)	EC1: High BRJ EC2: Medium BRJ EC3: Low BRJ EC4: PLA	EC1: BRJ 2 × 70 mL/day (12.9 mmol) (morning, evening)/13 weeks EC2: BRJ 70 mL/day (6.45 mmol) (evening)/13 weeks EC3: BRJ 35 mL/day (3.23 mmol) (evening)/13 weeks	Beet It Sports (James White Drinks, UK)	Blood Sampling, Urine Sampling	(EC1, EC2) Plasma NO_3_^−^ ↑ (EC3) Plasma NO_3_^−^ ↔ (EC1, EC3) Plasma NO_2_^−^ ↑ (EC2) Plasma NO_2_^−^ ↔ (EC1, EC2) Saliva NO_3_^−^ ↑ (EC3) Saliva NO_3_^−^ ↔ (EC1, EC2) Saliva NO_2_^−^ ↑ (EC3) Saliva NO_2_^−^ ↔ (EC1, EC2) Urine NO_3_^−^ ↑ (EC3, time points) Urine NO_3_^−^ ↔ (EC1, EC2, EC3) Urine NO_2_^−^ ↔
Fejes et al. [54]	M (NR) and W (NR) hypertension older (*n* = 15)	EC1: BRJ EC2: PLA	EC1: BRJ 2 × 70 mL/day (12.9 mmol) (NR)/4 weeks	Beet It (James White Drinks Ltd., Ipswich, UK)	Blood Sampling, Salivary nitrate, FBF, Clinic BP, Home BP	(3H post) Plasma NO_3_^−^, NO_2_^−^ ↑ Salivary NO_3_^−^, NO_2_^−^ ↑ FBF AUC ratio ↑ BP ↔ (4-week post) Plasma NO_3_^−^, NO_2_^−^ ↑ Salivary NO_3_^−^, NO_2_^−^ ↑ FBF AUC ratio ↔ BP ↔
Miller et al. [39]	M (*n* = 3) and W (*n* = 10) healthy middle-aged and older (*n* = 13)	EC1: BRJ EC2: PLA	EC1: BRJ 70 mL/day (6.1 mmol) (morning)/12 weeks	Beet It Sports (James White Drinks Ltd.; Ipswich, UK)	Blood Sampling	(90 min) Plasma NO_3_^−^ ↑ (90 min) Plasma NO_2_^−^ ↑ Fasting Plasma NO_3_^−^ ↑ Fasting Plasma NO_2_^−^ ↑ Plasma NO_2_^−^ Change Variability ↑
Vanhatalo et al. [51]	M (NR) and W (NR) healthy younger and older (*n* = 75)	EC1: BRJ (YN) EC2: BRJ (OLD) EC3: PLA (YN) EC4: PLA (OLD)	EC1: BRJ 2 × 70 mL/day (12.1 mmol) (morning and evening)/2 weeks EC2: BRJ 2 × 70 mL/day (12.1 mmol) (morning and evening)/2 weeks	Beetroot Juice	Tongue Scarping, Blood Sampling, Peripheral blood pressure, Central blood pressure, FMD	(EC2) MAP ↓ (EC1) MAP ↔ (EC2) ΔNO_2_^−^ ↑ (EC1) ΔNO_2_^−^ ↔ (EC2, EC4) ΔMAP & ΔNO_2_^−^ ↑ (EC1, EC3) ΔMAP & ΔNO_2_^−^ ↔ (EC2, EC4) NO_2_^−^ and oral microbes ↔ (EC1, EC3) NO_2_^−^ and oral microbes ↔
Wang et al. [52]	M (NR) and W (NR) healthy adults (*n* = 18)	EC1: BRJ	EC1: BRJ 270 mL/day (2.7 mmol) (morning and evening)/2 weeks	Red Beetroot Juice (Hartley Wintney, UK)	Stool Sampling	(Day3) Akkermansia muciniphila ↑ (Day3) Bacteroides fragilis ↓ (Day3, Day14) Total SCFAs ↑ (Day3, Day14) Butyric acid ↑ α- & β-diversity ↔

Note. Arrows indicate statistically significant differences as reported in the original studies (↑ increase, ↓ decrease); ↔ indicates no statistically significant difference.

### 4.2. Cardiovascular Health

Cardiovascular health encompasses a broad set of physiological processes, including blood pressure regulation, endothelial function, macrovascular and microvascular responses, peripheral and cerebral hemodynamics, and autonomic reflex activity. Acute findings are summarized in Table 3, with longer-term adaptations detailed in Table 4.

Across this literature, BRJ consistently increases circulating nitrate and nitrite, yet the magnitude and expression of downstream vascular effects depend heavily on baseline vascular status, task context, and the heterogeneity of outcome measures used across studies. Acute ingestion produces rapid hemodynamic adjustments largely attributed to increased NO formation. Meta-analytic estimates report systolic blood pressure (SBP) reductions of approximately 5–10 mmHg following a single dose [55]. These findings are supported by trials in healthy younger adults showing comparable decreases [56]. Improvement in endothelial responsiveness represents another frequently observed acute outcome. Increases in flow-mediated dilation (FMD) have been reported in pregnant women [57], while trials examining postprandial vascular function documented reduced vascular resistance [44]. Exercise studies further demonstrate elevated skeletal muscle blood flow [58], implying that BRJ may facilitate nutrient and oxygen delivery during physiological stress. Several studies additionally describe more stable systemic and cerebral hemodynamics following acute ingestion [59], although the precise mechanisms underlying these responses remain incompletely defined. Acute effects on autonomic responses appear more selective. Cardiovagal baroreflex sensitivity and indices of cerebral autoregulation generally remain unchanged [60,61]. In contrast, reduced peripheral chemoreflex sensitivity has been documented [62], suggesting that BRJ may influence oxygen-sensing pathways more readily than central autonomic control. Heart rate and HRV typically show minimal changes [60,61], and acute supplementation rarely alters structural vascular indices such as arterial stiffness [59].

Chronic BRJ ingestion has been associated with broader and more sustained cardiovascular adaptations, particularly in individuals with elevated baseline risk. Several meta-analyses report reproducible SBP reductions among hypertensive adults, with interventions lasting ≥14 days yielding more consistent effects [63,64]. In contrast, normotensive adults or older adults taking antihypertensive medications tend to exhibit smaller or more variable blood pressure changes [54,56]. Improvements in endothelial function have been documented across multiple chronic trials. Older adults showed increased FMD after one to four weeks of supplementation [65,66], and studies combining BRJ with dietary modification demonstrated enhanced NO-dependent microvascular regulation [48]. In individuals with mild cognitive impairment, supplementation increased cerebral microvascular responsiveness [67], suggesting that BRJ may exert more pronounced effects in populations with impaired vascular control. Extended supplementation also appears to affect peripheral vascular and oxygenation responses. A 12-week high-nitrate BRJ intervention was associated with concurrent improvements in lower-limb FMD, skeletal muscle microvascular function, and angiogenic potential [68]. Similar patterns have been observed in studies examining exercising muscle perfusion and tissue oxygen delivery [58,59,69]. These effects suggest the possibility that microvascular adaptation may precede structural modifications in larger vessels, although direct longitudinal evidence remains limited. Chronic intake also contributes to sustained changes in NO metabolism. Plasma nitrate and nitrite reliably increase over time [70], accompanied by shifts in oral nitrate-reducing bacteria and reductions in oxidative stress markers [71]. Despite these biochemical modifications, metrics such as HR, HRV, and arterial stiffness generally remain unchanged [65,71]. Selective autonomic effects have been observed, including reduced peripheral chemoreflex sensitivity [62], though these do not appear to generalize across broader autonomic domains. Across studies, chronic BRJ supplementation shows variable effects on cardiovascular physiology. Substantial improvements are typically observed in groups with compromised endothelial function or elevated cardiometabolic risk, while responses in younger, healthy, or already normotensive individuals are more modest. Accordingly, these findings should be interpreted in light of population-specific baseline risk and differences in study design, rather than as uniform effects across all groups.

### 4.3. Neuromuscular Function

Neuromuscular function includes strength, power, motor unit behavior, muscle excitability, fatigue resistance, and recovery capacity. Findings are summarized in Table 5.

Across studies, acute BRJ ingestion produces minimal changes in maximal voluntary contraction (MVC), rate of force development, or peak strength in sport climbers [72], tennis and basketball athletes [73], and female hockey players [74]. Similar patterns are reported in eccentric-exercise protocols, where MVC recovery and subjective soreness do not differ from placebo [26]. Power- and sprint-based performance outcomes also show limited responsiveness, although one study in semi-professional female rugby players documented an improvement in countermovement jump height [75]. Dose–response work adds nuance, suggesting that lower nitrate doses may influence the rate of torque development, whereas higher doses may affect peak torque [76]. Motor unit–level findings indicate that neural activation patterns remain largely unchanged following nitrate ingestion. Studies by Esen et al. show no alterations in motor unit firing rate, recruitment threshold, or motor unit potential area [77,78]. However, consistent reductions in motor unit potential duration suggest faster restoration of muscle fiber membrane conduction velocity, particularly during ischemic contractions, where BRJ improves the recovery of peripheral muscle excitability [77]. Functional indices of recovery—including MVC, countermovement jump, and pain thresholds—have improved in some studies even when biochemical markers of muscle damage (Creatine kinase (CK), *C*-reactive protein (CRP), IL-6) do not change [26,79].

Longer-term supplementation has produced few measurable changes in neuromuscular performance. Extended nitrate ingestion improved exercise tolerance in one trial but did not modify MVC, electromyography (EMG) amplitude, or fatigability [53]. Other multi-week interventions similarly report no changes in muscle strength, power, EMG-based activation, or motor unit recruitment strategies [80,81]. Across chronic trials, contractile properties and central neural control appear largely unaffected, and performance outcomes remain stable regardless of dose or supplementation duration. Taken across studies, BRJ’s neuromuscular effects appear most evident in contexts involving fatigue or recovery, where improvements in functional performance and muscle fiber conduction properties are occasionally observed. The degree to which these responses occur varies by supplementation dose, muscle group tested, and the specific physiological demands of the task being evaluated. Nevertheless, the available evidence remains limited in scope, and conclusions are primarily based on a relatively small number of heterogeneous studies.

**Table 3:** Acute effects of beetroot juice supplementation on cardiovascular health.

Reference	Participants	Experimental Conditions	Supplementation Protocol	Supplement Source	Variables	Results
Benjamim et al. [82]	W, systemic arterial hypertension postmenopausal adult (*n* = 14)	EC1: BRJ (1st D) EC2: BRJ	EC1: BRJ 2 × 70 mL/day (12.8 mmol) (morning)/1 week EC2: BRJ 70 mL/day (6.4 mmol) (morning)/1 week	Beet It Sports (James White Drinks Ltd., Ipswich, UK)	FMD, HRV, HR, BP, Blood Sampling	(EC1) SBP ↓ (EC2) SBP ↔ (EC1 vs. EC2) FMD ↑ (EC1 vs. EC2) HRV ↑ (EC1 vs. EC2) HR ↔ (EC1 & EC2) Plasma NO_3_^−^ ↑ (EC1 & EC2) Plasma NO_2_^−^ ↑
Curry et al. [59]	W, healthy adult (*n* = 10)	EC1: BRJ EC2: Orange Juice	EC1: BRJ 500 mL/day (12 mmol) (120 min before)/1 day	Beetroot Juice (CAJ Food Products, Inc., Fishers, IN, USA)	Electronically braked leg cycle ergometer (40 & 80% VO_2_peak for 5 min), BP, HR, Transcranial Doppler, CO	Blood NO ↑ SBP ↓ DBP & HR ↔ CAIx ↓ (Rest) CAIx ↔ PIx & RIx ↔
Chapman et al. [61]	M (*n* = 7) and W (*n* = 7) healthy adult (*n* = 14)	EC1: BRJ EC2: PLA	EC1: BRJ 500 mL/day (9.68 mmol) (180 min before)/1 day	Commercial BRJ (Biotta Beet Juice, Fishers, IN, USA)	Breathe CO_2_ for 5 min, Treadmill Walking Test, BP, Microcirculatory endothelial function	Renal/Segmental Artery blood velocity ↔ Vascular resistance ↔ Microcirculatory endothelial function ↔
Engan et al. [83]	M (*n* = 5) and W (*n* = 3) healthy adult (*n* = 8)	EC1: BRJ EC2: PLA	EC1: BRJ 70 mL/day (5 mmol) (120 min before)/1 day	Beetroot Juice (James White Drinks Ltd., Ipswich, UK)	Apnea Test, SpO_2_, HR, Spleen Maximal Diameters, Blood Sampling	Spleen Volume ↓ Hb Concentration ↑ Apnea Apleen Concentration ↔ Elevated Hb during Apnea ↔ Max Apnea Duration ↔ HR Drop during Apnea ↔ SpO_2_ Max ↔
Fejes et al. [71]	M (*n* = 10) and W (*n* = 5) older adults with medically treated hypertension (*n* = 15)	EC1: BRJ EC2: PLA	EC1: BRJ 70 mL/day (6.4 mmol) (morning)/1 day	Beet It (James White Drinks Ltd., Ipswich, UK)	Blood Sampling, FMD, BP	Plasma NO_2_^−^ ↑ Plasma NO_3_^−^ ↑ SBP, DBP ↔ Cardiovascular function ↔
Fuchs et al. [44]	M, obese, insulin-resistant (*n* = 16)	EC1: BRJ EC2: CON	EC1: BRJ 70 mL/day (4.8 mmol) (180 min before)/1 day	Beet It (James White Drinks Ltd., Ipswich, UK)	Glucose homeostasis, Conduit Artery Blood Flow, Vascular occlusion plethysmography, NIRS, BP	Vascular resistance ↓ Postprandial glucose ↔ Postprandial insulin ↔
Hayes et al. [84]	M, healthy adult (*n* = 15)	EC1: BRJ EC2: PLA	EC1: BRJ 140 mL/day (14 mmol) (morning)/1 day	Beet It Sport (James White Drinks Ltd., Ipswich, UK)	Blood Sampling, BP	Plasma NO_3_^−^ ↑ Plasma NO_2_^−^ ↑ SBP ↓ DBP ↓
Heredia-Martinez et al. [43]	M (*n* = 7) and W (*n* = 8) hemodialysis (*n* = 8) and healthy adult (*n* = 15)	EC1: BRJ(HD) EC2: BRJ EC3: PLA(HD) EC4: PLA	EC1: BRJ 70 mL/day (6.4 mmol) (NR)/1 day EC2: BRJ 70 mL/day (6.4 mmol) (NR)/1 day	Beetroot Juice (James White Drinks Ltd., Ipswich, UK)	Blood Sampling, Dialysate Sampling, Saliva Sampling, BP	(EC1 vs. EC2) Plasma NO_2_^−^& NO_3_^−^ ↑ (EC1 vs. EC2) Plasma NO_2_^−^ AUClast ↑ (EC1) Plasma NO_2_^−^ AUClast & t ½ ↑ (EC1 vs. EC2) BP & Plasma cGMP ↔ (EC1) Potassium & Safety and Tolerability ↔
Horiuchi et al. [85]	M, healthy adult (*n* = 12)	EC1: BRJ (Normoxia) EC2: BRJ (Hypoxia) EC3: PLA (Normoxia) EC4: PLA (Hypoxia)	EC1: BRJ 2 × 70 mL/day (12.9 mmol) (NR)/4 days EC2: BRJ 2 × 70 mL/day (12.9 mmol) (NR)/4 days	Beet It (James White Drinks, Ipswich, UK)	Blood Sampling, DCA, Internal carotid artery, SpO_2_, MAP, VE, PETCO_2_	(EC1, EC2 vs. EC3, EC4) Circulating NO_3_ ↑ (EC2, EC4 vs. EC1, EC3) DCA-RoR ↓ (EC1 vs. EC3) DCA-RoR ↔ (EC2 vs. EC4) DCA-RoR ↔ (EC2, EC4 vs. EC1, EC3) Internal carotid artery Diameter, Velocity, or Flow ↔
Jurga et al. [42]	M (*n* = 3) and W (*n* = 5) healthy adult (*n* = 8)	EC1: BRJ EC2: NIT	EC1: BRJ 2 × 70 mL/day (12.9 mmol) (60 min before)/1 day	Beet It Sport (James White Drinks)	Blood Sampling, Flow-mediated skin fluorescence	Plasma NOx ↑ Plasma Betaine/Choline ↑ Trimethylamine/ Trimethylamine *N*-oxide ↔
Kelly et al. [86]	M (*n* = 6) and W (*n* = 6) healthy older adult (*n* = 12)	EC1: BRJ EC2: PLA	EC1: BRJ 2 × 70 mL/day (9.6 mmol) (morning and mid-day)/1 day	Beet It Sports (James White Drinks, Ipswich, UK)	Moderate Treadmill Walking, Low/High-Intensity Kness Extension, 6MWT, Blood Sampling, BP, HR, Serial Sevens Subtraction Test	(EC1) SBP ↓ (EC1) DBP ↓ (EC1 & EC2) MAP ↓ (EC1) VO_2_ Mean Response Time ↓ 6MWT, Muscle Metabolism, Cognition, Brain Metabolism ↔
Londono-Hoyos et al. [87]	M (*n* = 14) and W (*n* = 2) HFpEF Patients (*n* = 16)	EC1: BRJ EC2: PLA	EC1: BRJ 140 mL/day (12.9 mmol) (120 min before)/1 day	Beet It Sport (James White Drinks Ltd., Ipswich, UK	Echo, Doppler ultrasound, BP	MAP, HR, CSA, Acute hemodynamic, Carotid Hydraulic Power, Carotid Power Penetration, Carotid Energy Penetration ↔ CO ↓
Pedrinolla et al. [67]	M (NR) and W (NR) younger (*n* = 10) and older (*n* = 10) and AD (*n* = 10) (*n* = 30)	EC1: BRJ (YN) EC2: BRJ (OLD) EC3: BRJ (AD) EC4: PLA (YN) EC5: PLA (OLD) EC6: PLA (AD)	EC1: BRJ 70 mL/day (6.4 mmol) (morning)/1 day EC2: BRJ 70 mL/day (6.4 mmol) (morning)/1 day EC3: BRJ 70 mL/day (6.4 mmol) (morning)/1 day	Beet It Sports (James White Drinks, Ipswich, UK)	Blood Sampling	(EC3 vs. EC1, EC2) Baseline Plasma NO_3_ ↓ (EC2 vs. EC1) Baseline Plasma NO_3_^−^ ↔ (EC1) Baseline Plasma NO_3_^−^ ↔ (EC3, EC1, EC2) Baseline Plasma NO_2_^−^ ↔ (EC3, EC1, EC2) Δ Plasma NO_3_^−^ ↑ (EC3, EC1, EC2) Δ Plasma NO_2_^−^ ↑ (EC3, EC1, EC2) Δ Vascular Responsiveness ↑ (EC3 vs EC1, EC2) Absolute Vascular Responsiveness ↓ (EC2 vs EC1) Absolute Vascular Responsiveness ↔ (EC1) Absolute Vascular Responsiveness ↔
Raubenheimer et al. [88]	M (*n* = 5) and W (*n* = 7) healthy older adults (*n* = 12)	EC1: High BRJ EC2: PLA	EC1: BRJ 140 mL/day (12.9 mmol) (morning)/1 day	Beet It (James White Drinks, UK)	BP, Flow cytometry, Thromboelastometry, Blood Sampling	(3 h EC1) SBP ↓, DBP ↓, MAP ↓ (3 h) Monocyte-platelet aggregates ↓ (3 h) CD11b+ granulocytes ↓ (3 h) Intrinsic pathway ↓ (6 h) Aprotinin-test ↓
Richards et al. [58]	M (*n* = 11) and W (*n* = 7) healthy young adults (*n* = 18)	EC1: BRJ-280 EC2: BRJ-210 EC3: PLA-280 EC4: PLA-210	EC1: BRJ 280 mL/day (16.8 mmol) (120 min before)/1 day EC2: BRJ 210 mL/day (12.6 mmol) (12 min before)/1 day	Beet It Sports (James White Drinks, Ipswich, UK)	FBF, Forced vital capacity, Forearm VO_2_, Handgrip Exercise	FBF ↑ Forced vital capacity ↑ Forearm VO_2_ ↑
Rogerson et al. [89]	M (NR) and W (NR) younger (*n* = 18) and older (*n* = 7) (*n* = 25)	EC1: BRJ (YN) EC2: BRJ (OLD) EC3: PLA (YN) EC4: PLA (OLD)	EC1: BRJ 70 mL/day (6.4 mmol) (NR)/1 day	Beetroot Juice (James White Drinks Company suffolk, UK)	Blood Sampling, Urine Sampling, BP, Microcirculatory endothelial function	(3 h, EC1 & EC2) NO_3_^−^ Metabolism ↑ (24 h, EC1) NO_3_^−^ Metabolism ↑ (EC1 & EC2) SBP, DBP ↔ (EC1 & EC2) Microcirculatory endothelial function ↔
Rowland et al. [53]	M, healthy adult (*n* = 12)	EC1: BRJ (MORN) EC2: BRJ (AFT) EC3: BRJ (EVE) EC4: PLA (MORN) EC5: PLA (AFT), EC6: PLA (EVE),	EC1: BRJ 2 × 70 mL/day (13 mmol) (morning)/1 day EC2: BRJ 2 × 70 mL/day (13 mmol) (mid-day)/1 day EC3: BRJ 2 × 70 mL/day (13 mmol) (evening)/1 day	Beet It (James White Drinks Ltd., Ipswich, UK)	Cycle Ergometer Severe-Intensity Exercise, TTE, Urine Sampling, Saliva Sampling, BP, PWV	(EC1 & EC2 & EC3) NO_3_^−^ Metabolism ↑ (Time) NO_3_^−^ Metabolism ↔ (EC1 & EC2 & EC3) Central SBP ↓ (Time) SBP ↔ (EC1 & EC2 & EC3 vs. Time) Brachial SBP ↔ (EC1 & EC2 & EC3 vs. Time) TTE ↔
Stanaway et al. [70]	M (*n* = 12) and W (*n* = 12) healthy younger and older (*n* = 24)	EC1: BRJ (YN) EC2: BRJ (OLD) EC3: PLA (YN) EC4: PLA (OLD)	EC1: BRJ 150 mL/day (10.5 mmol) (morning)/1 day EC2: BRJ 150 mL/day (10.5 mmol) (morning)/1 day	Beetroot Juice	Treadmill Walking (low-intensity aerobic exercise), Blood Sampling, Cognitive Measurements, BP, Choice Reaction Test, RVIP, Stroop test, Mood and Perceptual	(EC1 & EC2) SBP ↓ (EC2) DBP ↓ (EC1 & EC2 & EC3 & EC4) Stroop reaction time ↑ (EC1 & EC2) Plasma NO_3_^−^ ↑ (EC1 & EC2) Plasma NO_2_^−^ ↑ (EC1 & EC2) Cognitive Function ↑
Tyler et al. [45]	M (*n* = 2) and W (*n* = 5) T2D adult (*n* = 7)	EC1: BRJ EC2: PLA	EC1: BRJ 70 mL/day (6.4 mmol) (120 min before)/1 day	Beetroot Juice (James White Drinks, Ipswich., UK)	Blood Sampling, OGTT, BP	Glucose AUG ↓ ΔSVR ↓ Salivary NO_2_^−^ & NO_3_^−^ ↑ SBP & DBP ↔ HOMA-IR & QUICKI ↔
Vanhatalo et al. [51]	M (NR) and W (NR) healthy younger and older (*n* = 75)	EC1: BRJ (YN) EC2: BRJ (OLD) EC3: PLA (YN) EC4: PLA (OLD)	EC1: BRJ 2 × 70 mL/day (12.1 mmol) (morning and evening)/2 weeks EC2: BRJ 2 × 70 mL/day (12.1 mmol) (morning and evening)/2 weeks	Beetroot Juice	Tongue Scarping, Blood Sampling, Peripheral blood pressure, Central blood pressure, FMD	(EC2) MAP ↓ (EC1) MAP ↔ (EC2) ΔNO_2_^−^ ↑ (EC1) ΔNO_2_^−^ ↔ (EC2, EC4) ΔMAP & ΔNO_2_^−^ ↑ (EC1, EC3) ΔMAP & ΔNO_2_^−^ ↔ (EC2, EC4) NO_2_^−^ and oral microbes ↔ (EC1, EC3) NO_2_^−^ and oral microbes ↔
van der Avoort et al. [56]	M (*n* = 15) and W (*n* = 15) healthy adults (*n* = 30)	EC1: BRJ EC2: VEG	EC1: BRJ (NR) (6.5 mmol) (mid-day)/1 week	Beet It (James White Drinks, UK)	Blood Sampling, BP	Plasma NO_3_^−^/NO_2_^−^ ↑ SBP ↓ DBP ↓
Volino-Souza et al. [57]	W, Healthy pregnant (*n* = 12)	EC1: BRJ EC2: PLA	EC1: BRJ 140 mL/day (8.95 mmol) (150 min before)/1 day	Beetroot Juice (processed in-lab)	Urine Sampling, FMD, NIRS	FMD ↑ Urinary NO_3_^−^ ↑ StO_2_ ↔
Wei et al. [76]	M (*n* = 8) and W (*n* = 3) healthy adult (*n* = 11)	EC1: High BRJ EC2: Medium BRJ EC3: Low BRJ EC4: PLA	EC1: BRJ 210 mL/day (19.2 mmol) (150 min before)/1 day EC2: BRJ 140 mL/day (12.8 mmol) (150 min before)/1 day EC3: BRJ 70 mL/day (6.4 mmol) (150 min before)/1 day	Beet It (James White Drinks Ltd., Ipswich, UK)	BP, 5 min all-out maximal voluntary knee extension test	(EC1) SBP ↓ (EC2, EC3) SBP ↔ (EC1) DBP ↓ (EC1, EC2) DBP ↔ (EC1) MAP ↓ (EC1, EC2) MAP ↔ (EC1, EC2) Peak Torque ↑ (EC3) Peak Torque ↔ (EC1, EC2) Torque Impulse ↑ (EC1, EC2) Torque Impulse ↑ (EC3) RTD ↑ (EC1, EC2) RTD ↓ Whole Blood[S-nitrosothiol] ↑ RBC[S-nitrosothiol] ↑ (EC1, EC2, EC3) Muscle NO_3_^−^ ↑ (EC1, EC2, EC3) Muscle NO_2_^−^ ↔
Worley et al. [60]	M (*n* = 8) and W (*n* = 5) healthy adult (*n* = 13)	EC1: BRJ EC2: PLA	EC1: 500 m/day (12.1 mmol) (180 min before)/1 day	Beetroot Juice (Biotta, Carmel, IN, USA)	LBNP, Cerebral artery blood velocity, HR, BP, cBRS	VLF Coherence ↑ VLF Phase ↔ VLF Gain ↔ LF Coherence ↔ LF Phase ↔ LF Gain ↓ Static-LBNP ↔ cBRS ↔
Wylie et al. [35]	M, healthy adult (*n* = 10)	EC1: High BRJ EC2: Medium BRJ EC3: Low BRJ EC4: PLA	EC1: BRJ 280 mL/day (16.8 mmol) (150 min before)/1 day EC2: BRJ 140 mL/day (8.4 mmol) (150 min before)/1 day EC3: BRJ 70 mL/day (4.2 mmol) (150 min before)/1 day	Beet It (James White Drinks, Ipswich, UK)	Moderate- and severe-intensity cycling, BP, Blood Sampling	(EC1, EC2, EC3) Plasma NO_3_^−^ ↔ (EC1, EC2, EC3) Plasma NO_2_^−^ ↑ SBP ↓ DBP ↓ MAP ↓ (EC1, EC2, EC3) VO_2_ amplitude ↓ (EC1, EC2, EC3) Time to task ↑

Note. Arrows indicate statistically significant differences as reported in the original studies (↑ increase, ↓ decrease); ↔ indicates no statistically significant difference.

**Table 4:** Chronic effects of beetroot juice supplementation on cardiovascular health.

Reference	Participants	Experimental Conditions	Supplementation Protocol	Supplement Source	Variables	Results
Alharbi et al. [48]	M (*n* = 7) and W (*n* = 22) obesity mid-age and older (*n* = 29)	EC1: CR + BRJ EC2: CR	EC1: BRJ 70 mL/day (6.4 mmol) (morning)/2 weeks	Beet It (James White Ltd., Ashbocking, Suffolk, UK)	BP, REE, Handgrip Strength, Skin microvascular blood flow, IPAQ, Urine Sampling, Saliva Sampling	Average Microvascular Flux ↑ NO-dependent Endothelial Activity ↑ SBP ↓ Cognitive Function ↑ Oxidative Stress ↑ NO bioavailability ↑ Physical Strength ↑ Metabolic Adaptation ↔ Body composition ↔
Babateen et al. [47]	M (*n* = 24) and W (*n* = 38) overweight/obese older adults (*n* = 62)	EC1: High BRJ EC2: Medium BRJ EC3: Low BRJ EC4: PLA	EC1: BRJ 2 × 70 mL/day (12.9 mmol) (morning, evening)/13 weeks EC2: BRJ 70 mL/day (6.45 mmol) (evening)/13 weeks EC3: BRJ 35 mL/day (3.23 mmol) (evening)/13 weeks	Beet It Sports (James White Drinks, UK)	Blood Sampling, Urine Sampling	(EC1, EC2) Plasma NO_3_^−^ ↑ (EC3) Plasma NO_3_^−^ ↔ (EC1, EC3) Plasma NO_2_^−^ ↑ (EC2) Plasma NO_2_^−^ ↔ (EC1, EC2) Saliva NO_3_^−^ ↑ (EC3) Saliva NO_3_^−^ ↔ (EC1, EC2) Saliva NO_2_^−^ ↑ (EC3) Saliva NO_2_^−^ ↔ (EC1, EC2) Urine NO_3_^−^ ↑ (EC3, time points) Urine NO_3_^−^ ↔ (EC1, EC2, EC3) Urine NO_2_^−^ ↔
Babateen et al. [90]	M (*n* = 24) and W (*n* = 38) Overweight/Obese older adults (*n* = 62)	EC1: High BRJ, EC2: Medium BRJ, EC3: Low BRJ, EC4: PLA	EC1: BRJ 2 × 70 mL/day (12.9 mmol) (morning, evening)/13 weeks EC2: BRJ 70 mL/day (6.45 mmol) (evening)/13 weeks EC3: BRJ 35 mL/day (3.23 mmol)/13 weeks	Beetroot Juice (James White Company, UK)	BP, Endothelial Function	(EC2, EC3) SBP ↓ (EC1) SBP ↔ (EC1, EC2, EC3) DBP ↔ (EC1, EC2, EC3) Home BP ↔ (EC2, EC3) Endothelial function ↑ (EC1) Endothelial function ↔
Bock et al. [62]	M (*n* = 12) and W (*n* = 11) healthy younger and older (*n* = 23)	EC1: BRJ EC2: PLA	EC1: BRJ 190 mL/day (4 mmol) (NR)/4 weeks	Superbeets (HumanN Inc., Austin, TX, USA)	BP, HR, BP, cBRS, Peripheral chemoreceptor sensitivity	(EC1, post) Plasma NO_3_^−^ ↑ (EC2 post) Plasma NO_3_^−^ ↔ (EC1) SBP ↓ (EC2) SBP ↔ (EC1) MAP ↓ (EC2) MAP ↔ (EC1, EC2) HR ↔ (EC1) Chemoreflex sensitivity Ve ↓ (EC2) Chemoreflex sensitivity Ve ↔ (EC1, EC2) Chemoreflex sensitivity HR ↔ (EC1, EC2) cBRS ↔
Delgado Spicuzza et al. [66]	W, early & late-postmenopausal adult (*n* = 25)	EC1: BRJ (EPM) EC2: BRJ (LPM) EC3: PLA (EPM) EC4: PLA (LPM)	EC1: BRJ 70 mL/day (6.4 mmol) (NR)/1 week EC2: BRJ 70 mL/day (6.4 mmol) (NR)/1 week	Beet It Organic (James White Drinks Ltd., Ipswich, UK)	BP, HR, baPWV, Blood Sampling, Macrovascular Function	(Rest, EC1, EC2 vs. EC3, EC4) ΔFMD ↑ (Rest, 24 h pre) ΔFMD ↑ (EC1, EC2 vs EC3, EC4) SBP/DBP ↔ (24 h pre) SBP/DBP↔ (EC1, EC2 vs EC3, EC4) Plasma NO_3_^−^ ↑ (24 h pre) Plasma NO_2_^−^ ↑ (EC1, EC2 vs EC3, EC4) Plasma NO_2_^−^ ↔ (24 h pre) Plasma NO_2_^−^ ↔ (EC1, EC2 vs EC3, EC4) Ischemia–reperfusion injury FMD ↔ (24 h pre) Ischemia–reperfusion injury FMD ↔
Fejes, Pilat et al. [71]	M (*n* = 10) and W (*n* = 5) hypertension older (*n* = 15)	EC1: BRJ EC2: PLA	EC1: BRJ 2 × 70 mL/day (12.9 mmol) (morning and evening)/4 weeks	Beetroot Juice	BP, Urine Sampling, Fasting Blood	(4WK POST) oxLDL/NOx ratio ↓ GSH/GSSG ratio ↑ (vs. Baseline) High-sensitivity CRP ↓
Fejes et al. [50]	M (*n* = 10) and W (*n* = 5) hypertension older (*n* = 15)	EC1: BRJ EC2: PLA	EC1: BRJ 2 × 70 mL/day (12.9 mmol) (morning and evening)/4 weeks	Beet It (James White Drinks Ltd., Ipswich, UK)	BP, FBF, Blood Sampling, 24 h Ambulatory BP monitoring	Plasma NO_3_^−^ /NO_2_^−^ ↑ Salivary NO_3_^−^/ NO_2_^−^ ↑ Acetylcholine, Nitroglycerin ↔ FBF-AUC Ratio ↔ SBP, MAP ↔ Home BP ↔ 24 h Ambulatory BP monitoring ↔
Jones et al. [65]	M (NR) and W (NR) healthy older (*n* = 20)	EC1: BRJ EC2: PLA	EC1: BRJ 70 mL/day (6.4 mmol) (morning)/4 weeks	Beet It Sport (James White Drinks Ltd., Ipswich, UK)	BP FMD, Microvascular function, Tongue Scarping, Blood Sampling	FMD ↑ SBP ↓ DBP ↓ Microvascular function ↔
Osman et al. [69]	W, planning to conceive (*n* = 29)	EC1: BRJ EC2: BRJ + EXE EC3: EXE EC4: CON	EC1: BRJ 70 mL/day (6.4 mmol) (morning)/12 weeks EC2: BRJ 70 mL/day (6.4 mmol) (morning)/12 weeks	BRJ Supplementation Juice (James White Drinks Ltd., Ipswich, UK)	Resistance and Endurance exercise, BP, CO, TPR	(EC3) TPR ↓ (EC3) CO ↑ (EC2) DBP ↓ (EC2) CO ↑

Note. Arrows indicate statistically significant differences as reported in the original studies (↑ increase, ↓ decrease); ↔ indicates no statistically significant difference.

**Table 5:** Effects of beetroot juice supplementation on neurological function.

Reference	Participants	Experimental Conditions	Supplementation Protocol	Supplement Source	Variables	Results
Acute						
Berlanga et al. [72]	M, amateur sports climber (*n* = 10)	EC1: BRJ EC2: PLA	EC1: BRJ 70 mL/day (6.4 mmol) (150 min before)/1 day	Beet-It Pro Elite Shot (James White Drinks Ltd., Ipswich, UK)	Maximal Isometric Half Crimp Test, Pull-Up Failure Test, IHS, CMJ, SJ, Saliva Sampling	CMJ height ↔ SJ height ↔ IHS ↔ Pull-up test to failure ↔ Half crimp test ↔ Salivary NO_3_^−^ and NO_2_^−^ ↑
Clifford et al. [26]	M, healthy, recreationally active (*n* = 29)	EC1: High BRJ EC2: Low BRJ EC3: PLA	EC1: BRJ 250 mL/day (4 mmol) (morning and evening)/3 days EC2: BRJ 250 mL/day (2 mmol) (morning and evening)/3 days	Love Beets Super Tasty Beet Juice (Gs Fresh Ltd., Cambridgeshire, UK)	Drop Jump, MIVC, CMJ, PPT, CK	(EC1, EC2) MIVC recovery ↑ CMJ recovery ↑ CK ↓ PPT ↓
Garnacho-Castano et al. [41]	M, healthy with ≥ 2 years of crossfit experience (*n* = 12)	EC1: BRJ EC2: PLA	EC1: BRJ 140 mL/day (12.8 mmol) (180 min before)/1 day	Beet It (James White Drinks Ltd., Ipswich, UK)	Workout of the day Test (CrossFit), Blood Sampling, SpO_2_, CMJ	(1st) Number of Repetitions ↑ (2nd) Number of Repetitions ↔ Serum Cortisol SpO_2_ ↓ Muscle Fatigue ↓ Serum Testosterone ↔ Testosterone/cortisol Ratio ↔ Blood Lactate ↔
Lopez-Samanes et al. [91]	M, young basketball player (*n* = 10)	EC1: BRJ EC2: PLA	EC1: BRJ 140 mL/day (12.8 mmol) (180 min before)/1 day	Beet-It Pro Elite Shot (James White Drinks Ltd., Ipswich, UK)	Simulated Basketball game and Neuromuscular Performance Test	(EC1 vs. EC2) CMJ ↑ (EC1 vs. EC2) Sprint ↑ (EC1 vs. EC2) Handgrip ↑ (EC1 vs. EC2) Agility T-test ↑ match physical activity ↔
Lopez-Samanes et al. [73]	M, highly competitive tennis player (*n* = 13)	EC1: BRJ EC2: PLA	EC1: BRJ 70 mL/day (6.4 mmol) (180 min before)/1 day	Beet-It Pro Elite Shot (James White Drinks Ltd., Ipswich, UK)	RPE, Serve velocity test, CMJ, ISH, 5-0-5 agility, 10 m	Serve velocity test ↑ CMJ ↑ IHS ↑ 5-0-5 agility dominant ↓ 5-0-5 agility non-dominant side↓ 10 m ↓
Lopez-Samanes et al. [75]	W, semi-professional rugby player (*n* = 14)	EC1: BRJ EC2: PLA	EC1: BRJ 140 mL/day (12.8 mmol) (150 min before)/1 day	Beet-It Pro Elite Shot (James White Drinks Ltd., Ipswich, UK)	CMJ, IHS, 10 m and 30 m Sprint Test, Modified agility T-test, Bronco endurance test	CMJ ↑ IHS ↔ 10 m & 30 m Sprint ↔ Agility T-test ↔ Bronco endurance test ↔
Lopez-Samanes et al. [74]	W, elite field hockey player (*n* = 11)	EC1: BRJ EC2: PLA	EC1: BRJ 70 mL/day (6.4 mmol) (180 min before)/1 day	Beet-It Pro Elite Shot (James White Drinks Ltd., Ipswich, UK)	CMJ, IHS, 20 m-sprint, RSA	CMJ ↔ Handgrip ↔ 20 m Sprint ↔ RSA ↔ Match GPS metrics ↔
Rowland et al. [53]	M, healthy adult (*n* = 12)	EC1: BRJ (MORN) EC2: BRJ (AFT) EC3: BRJ (EVE) EC4: PLA (MORN) EC5: PLA (AFT) EC6: PLA (EVE)	EC1: BRJ 2 × 70 mL/day (13 mmol) (morning)/1 day EC2: BRJ 2 × 70 mL/day (13 mmol) (mid-day)/1 day EC3: BRJ 2 × 70 mL/day (13 mmol) (evening)/1 day	Beet It (James White Drinks Ltd., Ipswich, UK)	Cycle Ergometer Severe-Intensity Exercise, TTE, Urine Sampling, Saliva Sampling, BP, PWV	(EC1 & EC2 & EC3) NO_3_^−^ Metabolism ↑ (Time) NO_3_^−^ Metabolism ↔ (EC1 & EC2 & EC3) Central SBP ↓ (Time) SBP ↔ (EC1 & EC2 & EC3 vs. Time) Brachial SBP ↔ (EC1 & EC2 & EC3 vs. Time) TTE ↔
Tan et al. [81]	M, healthy and resistance-trained (*n* = 18)	EC1: High BRJ EC2: Medium BRJ EC3: Low BRJ EC4: PLA	EC1: BRJ 4 × 70 mL/day (24 mmol) (150 min before)/1 day EC2: BRJ 2 × 70 mL/day (12 mmol) (150 min before)/1 day EC3: BRJ 70 mL/day (6 mmol) (150 min before)/1 day	Beet It (James White Drinks Ltd., Ipswich, UK)	CMJ: 1 set × 5 reps (40% 1RM) Squat & Bench: 1 set × 3 reps (50% 1RM) + 1 set × 3 reps (75% 1RM), Liner position transducer, Blood Sampling, Brunel mood scale	CMJ ↔ Squat ↔ Bench Press ↔ (EC1, EC2, EC3) Plasma NO_3_^−^ ↔ (EC1, EC2, EC3) Plasma NO_2_^−^ ↑ Mood ↔
Wei et al. [76]	M (*n* = 8) and W (*n* = 3) healthy adult (*n* = 11)	EC1: High BRJ EC2: Medium BRJ EC3: Low BRJ EC4: PLA	EC1: BRJ 210 mL/day (19.2 mmol) (150 min before)/1 day EC2: BRJ 140 mL/day (12.8 mmol) (150 min before)/1 day EC3: BRJ 70 mL/day (6.4 mmol) (150 min before)/1 day	Beet It (James White Drinks Ltd., Ipswich, UK)	BP, 5 min all-out maximal voluntary knee extension test,	(EC1) SBP ↓ (EC2, EC3) SBP ↔ (EC1) DBP ↓ (EC1, EC2) DBP ↔ (EC1) MAP ↓ (EC1, EC2) MAP ↔ (EC1, EC2) Peak Torque ↑ (EC3) Peak Torque ↔ (EC1, EC2) Torque Impulse ↑ (EC1, EC2) Torque Impulse ↑ (EC3) RTD ↑ (EC1, EC2) RTD ↓ Whole Blood[S-nitrosothiol] ↑ RBC[S-nitrosothiol] ↑ (EC1, EC2, EC3) Muscle NO_3_^−^ ↑ (EC1, EC2, EC3) Muscle NO_2_^−^ ↔
Wylie et al. [35]	M, healthy adult (*n* = 10)	EC1: High BRJ EC2: Medium BRJ EC3: Low BRJ EC4: PLA	EC1: BRJ 280 mL/day (16.8 mmol) (150 min before)/1 day EC2: BRJ 140 mL/day (8.4 mmol) (150 min before)/1 day EC3: BRJ 70 mL/day (4.2 mmol) (150 min before)/1 day	Beet It (James White Drinks, Ipswich, UK)	Moderate- and severe-intensity cycling, BP, Blood Sampling	(EC1, EC2, EC3) Plasma NO_3_^−^ ↔ (EC1, EC2, EC3) Plasma NO_2_^−^ ↑ SBP ↓ DBP ↓ MAP ↓ (EC1, EC2, EC3) VO_2_ amplitude ↓ (EC1, EC2, EC3) Time to task (severe) ↑
Chronic						
Daab et al. [92]	M, semi-professional soccer player (*n* = 13)	EC1: BRJ EC2: PLA	EC1: 2 × 150 mL/day (8 mmol) (morning and evening)/1 week	Natural Beetroot Juice (Homemade, freshly processed)	Intermittent Shuttle Running, MVC, Q_tw,pot_, Voluntary activation	MVC ↓ Q_tw,pot_ ↓ Voluntary activation ↓
Esen et al. [78]	M (*n* = 10) and W (*n* = 6) healthy, physically active young adult (*n* = 16)	EC1: BRJ EC2: PLA	EC1: BRJ 2 × 70 mL/day (12.8 mmol) (morning and evening)/5 days	Beet-It Pro Elite Shot (James White Drinks Ltd., Ipswich, UK)	Isometric knee extension at 25% MVC with BFR	Plasma NO_2_^−^ ↑ MUP duration ↓ MUFR, MUP area ↔
Esen et al. [77]	M, healthy, recreationally active (*n* = 14)	EC1: BRJ EC2: PLA	EC1: BRJ 2 × 70 mL/day (12.8 mmol) (morning and evening)/5 days	Beet It (James White Drinks, Ipswich, UK)	Kness-Entensor Strength Test, Intramuscular EMG, Isometric contractions, Blood Sampling	MUP duration ↓ Area, MUFR ↔ Plasma NO_2_^−^ ↑
Munoz et al. [80]	M, semi-professional handball player (*n* = 12)	EC1: BRJ EC2: PLA	EC1: 70 mL/day (6.4 mmol) (mid-day)/3 days	Beet-It Pro Elite Shot (James White Drinks Ltd., Ipswich, UK)	IHS, CMJ, Throwing velocity, Agility T-test, RSA	IHS ↑ CMJ Height ↑ Throwing velocity ↔ Agility T-test↔ RSA ↔

Note. Arrows indicate statistically significant differences as reported in the original studies (↑ increase, ↓ decrease); ↔ indicates no statistically significant difference.

### 4.4. Brain Health and Cognitive Function

Neuromuscular outcomes reported in human trials (summarized in Table 5) show that acute BRJ ingestion rarely alters maximal strength, rate of force development, or high-intensity neuromuscular output across diverse populations.

Studies in sport climbers [72], tennis athletes [73], and female hockey players [74] consistently show unchanged MVC, handgrip force, pull-up performance, and short-duration power tasks. Eccentric-exercise protocols likewise report no differences in soreness, MVC recovery, or inflammatory markers [26]. Power- and sprint-based measures also tend to remain stable, although an increase in Countermovement jump (CMJ) height was observed in semi-professional female rugby players following acute supplementation [75]. Dose–response evidence further suggests that lower nitrate doses may enhance the rate of torque development, whereas higher doses influence peak torque [76], indicating that specific neuromuscular parameters may respond differently to nitrate availability. At the electrophysiological level, studies by Esen et al. demonstrate that motor unit firing rate, recruitment threshold, and MUP area remain unchanged following nitrate ingestion [77,78], yet repeated reductions in MUP duration point toward faster restoration of muscle fiber membrane conduction velocity. This effect is especially apparent under ischemic conditions, where nitrate improves the recovery of MUP duration and peripheral excitability without altering central activation strategies [77]. Functional recovery outcomes—such as restored MVC, improved CMJ, and increased pain thresholds—have been observed in some trials even when biochemical markers of muscle damage (CK, CRP, IL-6) do not change, suggesting that BRJ may support neuromuscular recovery through mechanisms not captured by standard damage biomarkers [26,79].

Chronic supplementation produces fewer measurable neuromuscular adaptations. Multi-week interventions show improved exercise tolerance in one study but little change in MVC, EMG amplitude, fatigability, or muscle contractile properties [53]. Similar null findings have been reported for strength, power, EMG-based activation, and motor unit recruitment after prolonged ingestion across different nitrate doses [80,81]. The available chronic evidence therefore suggests limited effects on central neuromuscular mechanisms or structural muscle function, although peripheral electrophysiological responses—such as faster restoration of membrane conduction—seen in acute studies may still be relevant depending on task demands. Together, the current literature indicates that BRJ does not consistently enhance maximal neuromuscular performance but may influence peripheral muscle excitability or functional recovery under specific physiological conditions, with responses varying according to muscle group, supplementation dose, and the characteristics of the activity performed.

## 5. Effects of Beetroot Juice and Exercise on Athletic Performance

**Figure 3:**
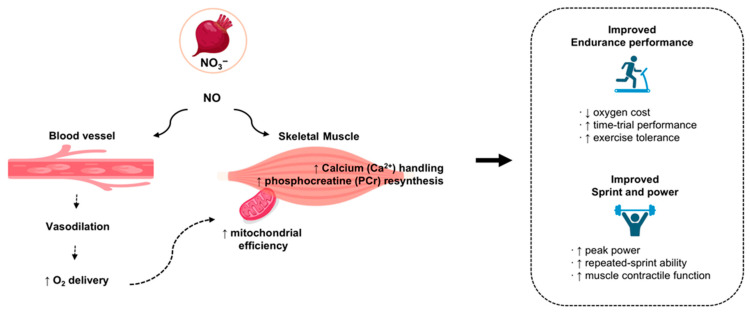
Nitrate-derived NO enhances vasodilation, mitochondrial efficiency, and Ca^2+^ handling, supporting improved endurance, sprint, and power performance. ↑ and ↓ indicate increases and decreases, respectively.

The mechanisms through which beetroot juice (BRJ) influences physiological responses to exercise—illustrated in Figure 3—form the basis for understanding its potential effects on athletic performance. Building on these mechanistic pathways, this section synthesizes findings from studies that administered BRJ acutely or over periods of sustained supplementation and evaluates how performance responses differ across endurance- based exercise, short-duration sprint and power tasks, and the intermittent, multidirectional demands typical of team sports. Because outcomes vary considerably with factors such as training status, exercise modality, and supplementation timing, the following subsections outline these performance domains separately to clarify when and under what conditions BRJ intake translates into measurable ergogenic effects (Table 6).

**Table 6:** Effects of beetroot juice supplementation on brain health and cognitive outcomes.

Reference	Participants	Experimental Conditions	Supplementation Protocol	Supplement Source	Variables	Results
Acute						
Curry et al. [59]	W, healthy (*n* = 10)	EC1: BRJ EC2: Orange Juice	EC1: BRJ 500 mL/day (12 mmol) (120 min before)/1 day	Beetroot Juice (CAJ Food Products, Inc., Fishers, IN, USA)	Electronically braked leg cycle ergometer (40 & 80% VO_2_peak for 5 min), BP, HR, Transcranial Doppler, CO	Blood NO ↑ SBP ↓ DBP & HR ↔ CAIx ↓ (Rest) CAIx ↔ PIx & RIx ↔
Horiuchi et al. [85]	M, healthy adult (*n* = 12)	EC1: BRJ (Normoxia), EC2: BRJ (Hypoxia) EC3: PLA (Normoxia) EC4: PLA (Hypoxia)	EC1: BRJ 2 × 70 mL/day (12.9 mmol) (NR)/4 days EC2: BRJ 2 × 70 mL/day (12.9 mmol) (NR)/4 days	Beet It (James White Drinks, UK)	Blood Sampling, DCA, Internal carotid artery, SpO_2_, MAP, VE, PETCO_2_	(EC1, EC2 vs. EC3, EC4) Circulating NO_3_^−^ ↑ (EC2, EC4 vs. EC1, EC3) DCA-RoR ↓ (EC1 vs. EC3) DCA-RoR ↔ (EC2 vs. EC4) DCA-RoR ↔ (EC2, EC4 vs. EC1, EC3) Internal carotid artery Diameter, Velocity, or Flow ↔
Londono-Hoyos et al. [87]	M (*n* = 14) and W (*n* = 2) HFpEF patients (*n* = 16)	EC1: BRJ EC2: PLA	EC1: BRJ 140 mL/day (12.9 mmol) (120 min before)/1 day	Beet It Sport (James White Drinks Ltd., UK)	Echo, Echo, BP	MAP, HR, CSA, Acute hemodynamic, Carotid Hydraulic Power, Carotid Power Penetration, Carotid Energy Penetration ↔ Cardiac Output ↓
Miraftabi et al. [93]	M, trained taekwondo athletes (*n* = 8)	EC1: BRJ-800 EC2: BRJ-400 EC3: PLA EC4: CON	EC1: BRJ 120 mL/day (12.9 mmol) (150 min before)/1 day EC2: BRJ 120 mL/day (6.4 mmol) (150 min before)/1 day	Red Beet Vinitrox Shot (Sponsor Ltd., Germany)	High-intensity Intermittent Exercise, PSTT, Multiple frequency speed of kick test, CMJ, Stroop Test, Finger-Prick Blood Sampling	PSTT, Multiple frequency speed of kick test ↔ (EC2, after PSTT) Stroop test ↑ CMJ Height ↔ HR ↔ RPE ↔ Blood Lactate ↔
Pedrinolla et al. [67]	M (NR) and W (NR) younger (*n* = 10) and older (*n* = 10) and AD (*n* = 10) (*n* = 30)	EC1: BRJ (YN) EC2: BRJ (OLD) EC3: BRJ (AD) EC4: PLA (YN) EC5: PLA (OLD) EC6: PLA (AD)	EC1: BRJ 70 mL/day (5.0 mmol) (morning)/1 day EC2: BRJ 70 mL/day (5.0 mmol) (morning)/1 day EC3: BRJ 70 mL/day (5.0 mmol) (morning)/1 day	Beet It Sports (James White Drinks, Ipswich, UK)	Blood Sampling	(EC3 vs. EC1, EC2) Baseline Plasma NO_3_^−^ ↓ (EC2 vs. EC1) Baseline Plasma NO_3_^−^ ↔ (EC1) Baseline Plasma NO_3_^−^ ↔ (EC3, EC1, EC2) Baseline Plasma NO_2_^−^ ↔ (EC3, EC1, EC2) Δ Plasma NO_3_^−^ ↑ (EC3, EC1, EC2) Δ Plasma NO_2_^−^ ↑ (EC3, EC1, EC2) Δ Vascular Responsiveness ↑ (EC3 vs EC1, EC2) Absolute Vascular Responsiveness ↓ (EC2 vs EC1) Absolute Vascular Responsiveness ↔ (EC1) Absolute Vascular Responsiveness ↔
Stanaway et al. [70]	M (*n* = 12) and W (*n* = 12) healthy younger and older (*n* = 24)	EC1: BRJ (YN) EC2: BRJ (OLD) EC3: PLA (YN) EC4: PLA (OLD)	EC1: BRJ 150 mL/day (10.5 mmol) (morning)/1 day EC2: BRJ 150 mL/day (10.5 mmol) (morning)/1 day	Beetroot Juice	Treadmill Walking (low-intensity aerobic exercise), Blood Sampling, Cognitive Measurements, BP, RVIP, Stroop test, Mood and Perceptual	(EC1, EC2) SBP ↓ (EC2) DBP ↓ (EC1, EC2, EC3, EC4) Stroop reaction time ↑ (EC1, EC2) Plasma NO_3_^−^ ↑ (EC1, EC2) Plasma NO_2_^−^ ↑ (EC1, EC2) Cognitive Function ↑
Thompson et al. [94]	M, healthy and active (*n* = 16)	EC1: BRJ EC2: PLA	EC1: BRJ 450 mL/day (5 mmol) (90 min before)/1 day	Beet It (James White Ltd., Ipswich, UK)	Electronically Braked leg Cycle Ergometer (RPE, Brunel mood scale, BP, Finger-Prick Blood Sampling, Cerebral NIRS, Muscle NIRS, Intramuscular EMG, RVIP) Stroop Test	Plasma Nitrate ↑ SBP ↓ VO_2_ ↔ HHb muscle ↓ HHb cerebral ↓ TTE ↑ RVIP ↔ RPE ↔ Mental Fatigue ↔ (pre-EXE) Lactate ↑
Wightman et al. [11]	M (*n* = 12) and W (*n* =28) healthy adult (*n* = 40)	EC1: BRJ EC2: PLA	EC1: 450 mL/day (5.5 mmol) (at test onset)	Beet It (James White Ltd., Ipswich, UK)	NIRS, Blood Sampling, RVIP	Plasma NO_3_^−^ ↑ Cognitive Performance ↑ (pre) Cerebral blood flow ↑ (post) Cerebral blood flow ↓ (Total Hb during RVIP) Cerebral blood flow ↓ Deoxy-Hb ↔ SBP/DBP ↔ HR ↔
Worley et al. [60]	M (*n* = 8) and W (*n* = 5) healthy adult (*n* = 13)	EC1: BRJ EC2: PLA	EC1: 500 m/day (12.1 mmol) (180 min before)/1 day	Beetroot Juice (Biotta, Carmel, IN, USA)	LBNP, Cerebral artery blood velocity, HR, BP, cBRS	VLF Coherence ↑ VLF Phase ↔ VLF Gain ↔ LF Coherence ↔ LF Phase ↔ LF Gain ↓ Static-LBNP ↔ cBRS ↔
Chronic						
Alharbi et al. [48]	M (*n* = 7) and W (*n* = 22) obesity mid-age and older (*n* = 29)	EC1: CR + BRJ EC2: CR	EC1: BRJ 70 mL/day (6.4 mmol) (morning)/2 weeks	Beet It (James White Ltd., Ashbocking, Suffolk, UK)	BP, REE, Handgrip Strength, Skin microvascular blood flow, IPAQ, Urine Sampling, Saliva Sampling	Average Microvascular Flux ↑ NO-dependent Endothelial Activity ↑ SBP ↓ Cognitive Function ↑ Oxidative Stress ↑ NO bioavailability ↑ Physical Strength ↑ Metabolic Adaptation ↔ Body composition ↔
Babateen et al. [47]	M (*n* = 24) and W (*n* = 38) overweight/obese older adults (*n* = 62)	EC1: High BRJ EC2: Medium BRJ EC3: Low BRJ EC4: PLA	EC1: BRJ 2 × 70 mL/day (12.9 mmol) (morning, evening)/13 weeks EC2: BRJ 70 mL/day (6.45 mmol) (evening)/13 weeks EC3: BRJ 35 mL/day (3.23 mmol) (evening)/13 weeks	Beet It Sports (James White Drinks, UK)	Blood Sampling, Urine Sampling	(EC1, EC2) Plasma NO_3_^−^ ↑ (EC3) Plasma NO_3_^−^ ↔ (EC1, EC3) Plasma NO_2_^−^ ↑ (EC2) Plasma NO_2_^−^ ↔ (EC1, EC2) Saliva NO_3_^−^ ↑ (EC3) Saliva NO_3_^−^ ↔ (EC1, EC2) Saliva NO_2_^−^ ↑ (EC3) Saliva NO_2_^−^ ↔ (EC1, EC2) Urine NO_3_^−^ ↑ (EC3, time points) Urine NO_3_^−^ ↔ (EC1, EC2, EC3) Urine NO_2_^−^ ↔
Babateen et al. [95]	M (*n* = 24) and W (*n* = 38) overweight/obese older adults (*n* = 62)	EC1: High BRJ EC2: Medium BRJ EC3: Low BRJ EC4: PLA	EC1: BRJ 2 × 70 mL/day (12.9 mmol) (morning, evening)/13 weeks EC2: BRJ 70 mL/day (6.45 mmol) (evening)/13 weeks EC3: BRJ 35 mL/day (3.23 mmol)/13 weeks	Beetroot Juice (James White Company, Ashbocking, Suffolk, UK)	qNIRS, Cognitive Function	Cognitive Function ↔ Cerebral blood flow ↔ (EC3) Plasma NO_3_^−^ ↔ (EC1, EC3) Plasma NO_2_^−^ ↑
Kelly et al. [86]	M (*n* = 6) and W (*n* = 6) healthy older adult (*n* = 12)	EC1: BRJ EC2: PLA	EC1: BRJ 2 × 70 mL/day (9.6 mmol) (morning and mid-day)/1 day	Beet It Sports (James White Drinks, UK)	Moderate Treadmill Walking, Low/High-Intensity Kness Extension, 6MWT, Blood Sampling, BP, HR, Serial Sevens, Subtraction Test	(EC1) SBP ↓, (EC1) DBP ↓ (EC1 & EC2) MAP, (EC1) VO _2_ Mean Response Time ↓ 6MWT, Muscle Metabolism, Cognition, Brain Metabolism ↔

Note. Arrows indicate statistically significant differences as reported in the original studies (↑ increase, ↓ decrease); ↔ indicates no statistically significant difference.

### 5.1. Endurance Athletes

Research on endurance performance shows the most consistent improvements with BRJ supplementation, reflecting its influence on oxygen cost, exercise economy, and tolerance to prolonged or high-intensity exertion. Findings summarized in Table 7 (acute) and Table 8 (chronic) demonstrate that BRJ can enhance aerobic performance across cycling, running, rowing, swimming, and other endurance-based activities.

Meta-analytic evidence indicates reductions in the oxygen cost of submaximal exercise, improved mitochondrial and contractile efficiency, and enhanced NO-mediated vasodilation, which together contribute to better exercise economy [96]. In trained cyclists, acute supplementation shortened 4 km and 16.1 km time-trial performance by 2.7–2.8% and increased the ratio of power output to oxygen consumption (PO/VO_2_) [14]. Similar benefits have been documented in rowing, where a single BRJ dose improved 2000 m time-trial performance and increased VO_2_max in master rowers [97], and in alpine skiing, where slalom completion time improved following supplementation [98]. Running performance also shows positive responses, with improvements of ~1.9% in 1500 m time trials following acute ingestion [99]. BRJ also demonstrates clear benefits for exercise economy and tolerance during sustained or intermittent efforts. In low-fitness individuals, four days of supplementation reduced submaximal oxygen consumption at 45% and 60% VO_2_max, whereas high-fitness individuals showed no change [100]. Competitive swimmers exhibited reduced aerobic energy cost and higher workloads at the anaerobic threshold after six days of BRJ ingestion [101]. During prolonged moderate-intensity cycling, BRJ preserved elevated plasma nitrite concentrations and attenuated the rise in oxygen uptake, reflecting improved submaximal economy when taken both before and during exercise [102]. Improvements in time to exhaustion (TTE) have been consistently reported across populations. Adolescents with obesity showed a ~23% increase in TTE after six days of supplementation, accompanied by reductions in the VO_2_ slow component [103]. Intermittent endurance capacity responds similarly, with single-dose BRJ increasing Yo-Yo IR1 performance [13] and three-day supplementation increasing total work and number of bouts completed during supramaximal intermittent cycling [104]. Cardiorespiratory variables may also improve under certain conditions; in female endurance athletes, acute intake increased VO_2_max by ~4.8% and improved ventilatory efficiency [105], although other studies in rowers observed performance benefits without changes in ventilatory responses [97]. Performance gains are not universal, particularly among elite endurance athletes whose physiological systems may already operate near their maximal NO-related capacity [106]. In highly trained 1500 m runners, BRJ supplementation did not affect running economy or time-trial performance [107], and in competitive swimmers, acute ingestion did not enhance repeated interval performance [101]. These heterogeneous responses indicate that the ergogenic effects of BRJ are more likely to emerge in athletes whose oxygen cost, mitochondrial efficiency, or NO-mediated vasodilation retain greater capacity for improvement.

### 5.2. Sprint and Power Athletes

The effects of BRJ supplementation on sprint and power performance are mixed and strongly context dependent, with improvements appearing in certain explosive tasks but not consistently expressed across protocols or athlete groups. In short-duration maximal efforts, several studies have reported clear benefits. A single dose of BRJ increased peak and mean power and shortened the time to reach peak power during a 30 s all-out Wingate sprint in resistance-trained men [108]. Maximal cycling sprints lasting 3–4 s have shown ~6% increases in peak power and higher optimal pedaling rates following BRJ ingestion [109]. In physically active women, supplementation improved CMJ height, power output, and barbell velocity during back squats, along with better repetition performance at 75% 1RM [110]. Similar improvements in explosive strength and Special Judo Fitness Test (SJFT) scores have been observed in elite adolescent judo athletes [111], suggesting that very brief, Type II fiber–dominant efforts may be particularly responsive to BRJ. Benefits have also been documented during repeated high-intensity resistance tasks. In healthy men, BRJ increased mean and peak power during sets performed at 60–80% 1RM, accompanied by higher barbell velocity and less power decline across repetitions [108]. Recovery-related outcomes demonstrate a similar pattern. After exercise-induced muscle damage, supplementation reduced muscle soreness and thigh swelling and helped restore static muscular endurance [112]. A systematic review has likewise shown improvements in muscle function recovery—especially MVC and CMJ—within 24–72 h [79]. These findings may reflect enhanced perfusion, improved metabolic efficiency, and NO-mediated support for excitation–contraction processes. At the same time, several well-controlled trials show minimal or no benefit. In some resistance-trained or sport-trained populations, BRJ has failed to meaningfully change peak force, movement velocity, repetition performance, or maximal isometric strength. These neutral outcomes are often observed in highly trained athletes whose neuromuscular systems are already operating near their mechanical and metabolic limits, leaving less opportunity for supplementation to influence contractile performance. BRJ appears to influence discrete components of sprint and power performance—particularly maximal power expression over very short durations, the maintenance of power during repeated efforts, and aspects of recovery from muscle-damaging activity—while showing limited effects on traditional strength measures or tasks requiring prolonged neuromuscular output. The available findings for acute and chronic protocols are summarized in Table 9 and Table 10.

### 5.3. Team-Sport Athletes

Team sports impose a distinct set of physiological and technical demands—continuous alternation between aerobic and anaerobic efforts, frequent changes in direction, contact situations, and decision-making under fatigue. These characteristics make the performance responses to BRJ supplementation different from those observed in isolated sprint or power protocols. Studies relevant to team sports from the current evidence base are summarized in Table 11, and their main findings are outlined below.

Across several investigations, repeated-sprint performance itself did not consistently improve, yet partial benefits appeared in recovery-related outcomes and in the preservation of function under fatigue. For example, in a study examining recovery between sprint bouts, BRJ ingestion facilitated better restoration of muscle function and reduced soreness, even though sprint performance per se remained unchanged [112]. In team-sport athletes such as basketball players, acute BRJ supplementation did not improve neuromuscular performance or match-play activity, supporting the notion that improvements in explosive actions may be context-dependent [91].

Some studies have reported performance enhancements under more specific conditions. In an intermittent-exercise model relevant to team sports, 7 days of BRJ supplementation increased total work performed during an 80 min protocol and helped maintain cognitive reaction speed in later stages, indicating protection of decision-making capacity under fatigue [113]. By contrast, protocols involving very high-intensity efforts with minimal recovery demonstrate a different response pattern. In an intermittent sprint task consisting of repeated 8 s all-out sprints with 30 s active recovery, a single dose of BRJ resulted in fewer completed sprints and lower total work, while mean and peak power, heart rate, blood lactate, and perceived exertion were unaffected [114]. BRJ supplementation in team-sport athletes appears to offer benefits in selected areas—such as recovery of muscle function, attenuation of soreness, maintenance of work output during prolonged intermittent exercise, and preservation of cognitive performance under fatigue—while showing inconsistent effects on key sport-specific outcomes including sprint speed, repeated-sprint ability, change-of-direction performance, and high-speed running. These variable responses likely reflect interactions among exercise structure, technical demands, recovery duration, athlete training status, and supplementation parameters, which together shape whether and how performance improvements emerge in team-sport environments.

**Table 7:** Acute effects of beetroot juice supplementation on endurance performance.

Reference	Participants	Experimental Conditions	Supplementation Protocol	Supplement Source	Variables	Results
Ahmadpour et al. [98]	M, expert alpine Skiers (*n* = 10)	EC1: BRJ EC2: PLA	EC1: BRJ 220 mL/day (8.9 mmol) (150 min before)/1 day	Beetroot Juice (Zarghan, Lepoi Fars, Shiraz, Iran)	90 s box jump, Hexagonal agility jump, wall-sit test, slalom runs at 15 min intervals	90 s box jump ↑ Wall-sit endurance ↑ Hex jump time ↓ Slalom run time ↔
Aucouturier et al. [104]	M, physically Active (*n* = 12)	EC1: BRJ EC2: PLA	EC1: BRJ 500 mL/day (9.3 mmol) (120 min before)/3 days	Beetroot juice (Pajottenlander, Belgium)	15 s at 170% MAP + 30 s passive rest repeated to exhaustion (Number of repetitions to exhaustion, RBC, VO_2_, lactate, MVC)	Number of repetitions to exhaustion ↑ RBC ↑ VO_2_, lactate, MVC ↔
Garnacho-Castaño et al. [97]	M, master rowers (*n* = 10)	EC1: BRJ EC2: PLA	EC1: BRJ 140 mL/day (12.8 mmol) (180 min before)/1 day	Beet it (James White Drinks Ltd., Ipswich, UK)	2000 m rowing ergometer test (TT time, mean power output, strokes/min, meters/stroke, VO_2_ (absolute & relative), HR max & mean, VE·VCO_2_^−1^ slope, blood lactate, SpO_2_, RPE)	TT time ↓ VO_2_ (absolute & relative) ↑ HR max & HR mean ↑ VE·VCO_2_^−1^ slope ↔ Blood lactate ↔ SpO_2_ ↔ RPE ↔ Mean power output ↔ Strokes/min ↔ Meters/stroke ↔
Lansley et al. [14]	M, competitive cyclists (*n* = 9)	EC1: BRJ EC2: PLA	EC1: BRJ 500 mL/day (6.2 mmol) (180 min before)/1 day, EC2: BRJ 500 mL/day (0.0047 mmol) (180 min before)/1 day	Beet It (James White Drinks Ltd., UK)	4 km and 16.1 km cycling time-trial (TT time, power output, VO_2_, HR, RPE)	TT time ↓ Power output ↑ VO_2_ ↓ HR ↔ RPE ↔
Moreno et al. [115]	M (*n* = 7) and W (*n* = 6) competitive swimmers (*n* = 13)	EC1: BRJ EC2: PLA	EC1: BRJ 70 mL/day (6.4 mmol) (180 min before)/1 day EC2: BRJ 70 mL/day (0.04 mmol) (180 min before)/1 day	Beet-It-Pro Elite Shot (James White Drinks Ltd., Ipswich, UK)	6 × 100 m repeated maximal-effort swimming test (front-crawl) with 7 min rest between sprints (Time per 100 m, RPE, TQR scale, blood lactate concentration	Time per 100 m ↔ RPE ↓ TQR ↑ Blood lactate ↔
Moreno-Heredero et al. [116]	M (*n* = 9) and W (*n* = 9) competitive swimmers (*n* = 18)	EC1: BRJ EC2: PLA	EC1: BRJ 70 mL/day (6.4 mmol) (120 min before)/1 day EC2: PLA 70 mL/day (0.04 mmol) (120 min before)/1 day	Beet-It-Pro Elite Shot; James White Drinks Ltd., Ipswich, UK	6 × 100 m front-crawl interval swimming with 5 min rest between repetitions (time per 100 m, RPE, TQR, HR, blood lactate)	Time per 100 m ↔ RPE ↔ TQR ↔ HR ↔ Blood lactate ↔
Zhang et al. [117]	W, recreationally active young (*n* = 13)	EC1: High BRJ EC2: Low BRJ EC3: PLA	EC1: BRJ 140 mL/day (12.9 mmol) (150 min before)/1 day EC2: BRJ 140 mL/day (6.45 mmol) (150 min before)/1 day	Beet It Sport (James White Drinks Ltd., Suffolk, England, UK)	High intensity interval training (PPO, HR, RPE, Plasma NO_3_^−^·NO_2_^−^)	(EC1, EC2) PPO ↔ TTE ↔ HR ↓ RPE ↓ Plasma NO_3_^−^, NO_2_^−^ ↑

Note. Arrows indicate statistically significant differences as reported in the original studies (↑ increase, ↓ decrease); ↔ indicates no statistically significant difference.

**Table 8:** Chronic effects of beetroot juice supplementation on endurance performance.

Reference	Participants	Experimental Conditions	Supplementation Protocol	Supplement Source	Variables	Results
Boorsma et al. [107]	M, elite 1500 m runners (*n* = 8)	EC1: BRJ EC2: PLA	EC1: BRJ 210 mL (19.5 mmol NO_3_^−^) 150 min before on days 1 and 8 + 140 mL/day (13.0 mmol NO_3_^−^) on days 2–7 (8 days total). EC2: PLA 140 mL (0.065-mmol NO_3_^−^) on 2–7 day	Beet It Sport (James White Drinks, Ipswich, UK)	Submaximal treadmill running at 50, 65, 80% VO_2_peak (VO_2_, HR, etc.), 1500 m time-trial performance, plasma [NO_3_^−^].	Plasma NO_3_^−^ ↑ submax VO_2_ ↔ HR ↔ 1500 m TT time ↔
Carriker et al. [118]	M, high fit (*n* =6) and low Fit (*n* =5), (*n* =11)	EC1: BRJ EC2: PLA	EC1: BRJ 70 mL/day (6.2 mmol) (150 min before)/4 days	Beet it (James White Drinks, Ltd., Ipswich, UK)	Submaximal treadmill bouts at 45%, 60%, 70%, 80%, 85% of VO_2_max (VO_2_, HR, RER, RPE)	VO_2_ ↓ VO_2_ ↔ HR ↔ RER ↔ RPE ↔
Pinna et al. [119]	M, master swimmers (*n* = 14)	EC1: BRJ, EC2: PLA	EC1: BRJ 500 mL/day (5.5 mmol) (a set time each day)/6 days	Reed Beet Juice, (Aureli, Ortucchio, Italy)	Control Swimming Test (VO_2_, VCO_2_, VE, Aerobic exergy expenditure)	Aerobic energy expenditure ↓ VO_2_ ↔ VCO_2_ ↔ VE ↔
Rasica et al. [103]	M (*n* = 2) and W (*n* = 8) obese adolescents (*n* = 10)	EC1: BRJ EC2: PLA	EC1: BRJ 2 × 70 mL/day (10 mmol) (a set time each day)/6 days	Beet It (James White Drinks, Ipswich, UK)	Moderate-intensity endurance (6 min × 2), Severe-intensity endurance (to exhaustion) (MRT, primary VO_2_, VO_2_ gain, VO_2_ slow component, End-exercise VO_2_, HR, lactate, TTE)	MRT ↓ VO_2_ ↔ VO_2_ gain ↔ VO_2_ slow component ↓ End-exercise VO_2_ ↔ HR ↔ Lactate ↔ TTE ↑
Tan et al. [102]	M, recreationally active (*n* = 12)	EC1: BRJ + BRJ EC2: BRJ + PLA EC3: PLA + PLA	EC1: BRJ 2 × 70 mL/day (12.8 mmol) (150 min before, 60 min after)/3 days EC2: BRJ 2 × 70 mL (12.8 mmol NO_3_^−^) (150 min before)/3 days	Beet it (James White Drinks, Ipswich, UK)	Moderate-intensity endurance Cycling (2 × 15 min) (VO_2_, VO_2_ drift, O_2_ cost, HR, RPE, Lactate, Plasma NO_2_^−^)	(EC1) VO_2_ drift ↓ O_2_ cost ↓ Mean VO_2_ ↔ HR ↔ RPE ↔ Lactate ↔ Muscle glycogen ↔ (EC1, EC2) Muscle ATP ↓ (EC1, EC2) Plasma NO_2_^−^ ↑

Note. Arrows indicate statistically significant differences as reported in the original studies (↑ increase, ↓ decrease); ↔ indicates no statistically significant difference.

**Table 9:** Acute effects of beetroot juice supplementation on sprint and power performance.

Reference	Participants	Experimental Conditions	Supplementation Protocol	Supplement Source	Variables	Results
Cuenca et al. [108]	M, healthy resistance-trained (*n* = 15)	EC1: BRJ EC2: PLA	EC1: BRJ 70 mL/day (6.4 mmol) (180 min before)/1 day	Beet-It-Pro Elite Shot (Beet IT; James White Drinks Ltd., Ipswich, UK)	30 s Wingate sprint (Peak power, Mean power, Time-to-peak, fatigue index, CMJ, EMG, Lactate, HR, RPE)	Peak power ↑ Mean power ↑ Time-to-peak ↓ Fatigue index ↔ CMJ ↔ EMG fatigue ↔ Lactate/HR/RPE ↔
Demirli et al. [111]	M, recreational adolescent judokas (*n* = 35)	EC1: BRJ EC2: PLA	EC1: BRJ 140 mL/day (12.8 mmol) (120 min before)/1 day	Beet It Sport (James White Drinks Ltd., Suffolk, England, UK)	4 min randori + Sargent jump + Back strength + Handgrip + SJFT (throws, index, 1 min HR)	Jump height ↑ Back strength ↑ Handgrip strength SJFT throws ↑ 1 min post-SJFT HR ↓ SJFT index ↓ Immediate post HR ↔ RPE ↔
Esen et al. [120]	M, trained rugby players (*n* = 12)	EC1: BRJ EC2: PLA	EC1: BRJ 140 mL/day (12.8 mmol) (120 min before)/1 day EC1: BRJ 140 mL/day (0.08 mmol) (180 min before)/1 day	Beet It (James White Drinks Ltd., Ipswich, UK)	YYIR1 (distance, HR, RPE, lactate, CMJ, IMTP, blood pressure)	Distance ↔ HR ↔ Lactate ↔ RPE ↔, CMJ ↔ IMTP ↔ BP ↔
Jurado-Castro et al. [110]	W, physically active athletes (*n* = 14)	EC1: BRJ EC2: PLA	EC1: BRJ 70 mL/day (6.4 mmol) (120 min before)/1 day	Beet It Sport (James White Drinks Ltd., Suffolk, England, UK)	Isometric mid-thigh pull test, CMJ, back-squat velocity test, NIRS (IMTP peak force, IMTP RFD, CMJ height, CMJ peak power, CMJ peak velocity, squat mean propulsive velocity, squat peak velocity)	IMTP peak force ↑ IMTP RFD ↑ CMJ peak power ↑ CMJ height/peak velocity/peak force ↔ 50% 1RM_Squat velocity ↑ 75% 1RM_Squat velocity ↔
Lopez-Samanes et al. [91]	M, young basketball players (*n* = 10)	EC1: BRJ EC2: PLA	EC1: BRJ 140 mL/day (12.8 mmol) (180 min before)/1 day	Beet It Sport (James White Drinks Ltd., Suffolk, England, UK)	Simulated 5-on-5 match + battery test (CMJ, 10 m sprint, 20 m sprint, handgrip test, agility T-test)	CMJ ↔ 10 m sprint time ↔ 20 m sprint time ↔ Handgrip strength ↔ Agility time ↔
Lopez-Samanes et al. [73]	M, well-trained tennis players (*n* = 13)	EC1: BRJ EC2: PLA	EC1: BRJ 70 mL/day (6.4 mmol) (180 min before)/1 day	Beetroot juice (Beet IT; James White Drinks Ltd., Ipswich, UK)	CMJ, Handgrip, 5–0–5 agility, 10 m sprint, Tennis serve test (CMJ height, CMJ, handgrip strength, agility time, 10-m sprint time, serve velocity, HR, RPE)	CMJ ↔ Handgrip ↔ Agility time ↔ 10 m sprint ↔ Serve velocity ↔ HR/RPE ↔
Lopez-Samanes et al. [75]	W, semi-pro rugby players (*n* = 14)	EC1: BRJ EC2: PLA	EC1: BRJ 140 mL/day (12.8 mmol) (150 min before)/1 day EC2: PLA 140 mL/day (0.08 mmol) (150 min before)/1 day	Beet-It-Pro Elite Shot, James White Drinks Ltd., Ipswich, UK	CMJ, handgrip strength, 10 m sprint, 30 m sprint, modified agility T-test, Bronco test (CMJ, handgrip strength, 10-m sprint time, 30-m sprint time, agility time, Bronco time)	CMJ↑ Handgrip strength ↔ 10 m sprint time ↔ 30 m sprint time ↔ Agility time ↔ Bronco time ↔
López-Samanes et al. [74]	W, elite female field hockey players (*n* =11)	EC1: BRJ EC2: PLA	EC1: BRJ 70 mL/day (6.4 mmol) (150 min before)/1 day EC2: BRJ 70 mL/day (0 mmol) (150 min before)/1 day	Beet-It-Pro Elite Shot, James White Drinks Ltd., Ipswich, UK	CMJ, isometric handgrip strength, 20 m sprint, repeated sprint ability test, simulated match play (CMJ height, handgrip strength, 20-m sprint time, repeated sprint ability mean time, total distance, high-intensity distance)	CMJ height ↔ Handgrip strength ↔ 20 m sprint time ↔ Repeated sprint ability mean time ↔ Total distance ↔ High-intensity distance ↔
Montalvo-Alonso et al. [121]	M, resistance-trained (*n* = 13)	EC1: CAF EC2: BRJ EC3: CAF + BRJ EC4: PLA	EC2: BRJ 70 mL/day (6.5 mmol) (180 min before)/1 day EC4: BRJ 70 mL/day (0.04 mmol) (150 min before)/1 day	Beet IT (James White Drinks Ltd., Ipswich, UK)	Back squat & bench press strength/power test + endurance at 65% 1RM (Back-squat & bench-press 25/50/75/90/100%1RM mean & peak velocity/power; endurance reps, mean velocity, mean power; plus, load)	(EC1, EC2, EC3) Back squat mean velocity & mean power ↑ Bench press ↔ (EC1, EC2, EC3) Endurance (65% 1RM back squat) ↑ Endurance (bench press) ↔
Rimer et al. [109]	M, trained athletes (*n* = 13)	EC1: BRJ EC2: PLA	EC1: BRJ 70 mL/day (11.2 mmol) (150 min before)/1 day	BEET It Sport (James White Drinks Ltd., Ipswich, UK)	3–4 s maximal sprint, 30 s maximal isokinetic cycling test (Maximal power, Optimal cadence, 30-s peak power, total work, fatigue index)	Maximal power ↑ Optimal cadence ↑ 30 s peak power ↔ Total work ↔ Fatigue index ↔
Shannon et al. [99]	M, runners or triathletes (*n* = 8)	EC1: BRJ + 1500 m TT EC2: PLA + 1500 m TT EC3: BRJ + 10,000 m TT EC4: PLA + 10,000 m TT	EC1, EC3: BRJ 140 mL/day (12.5 mmol) (180 min before)/1 day EC2, EC4: PLA 140 mL/day (0.01 mmol) (180 min before)/1 day	Beet It (James White Ltd., Ipswich, UK)	1500 m TT, 10,000 m TT (time-to-complete, post-exercise blood lactate)	(EC1) 1500 m TT time ↓, Lactate ↑ (EC3) 10,000 m TT time ↔ Lactate ↔
Wang et al. [122]	M, college bodybuilders (*n* = 16)	EC1: BRJ EC2: PLA	EC1: BRJ 250 mL/day (12.48 mmol) (150 min before)/1 day EC2: BRJ 250 mL/day (0.0005 mmol) (150 min before)/1 day	beetroot powder (Felicific Inc., New York, NY, USA)	Isometric circuit endurance test targeting elbow flexors, core muscles, forearm muscles, knee extensors (MVIC × 70% until fatigue) (MVIC peak torque, serum NO_3_^−^, NO_2_^−^, endurance, HR, RPE, lactate, RMS EMG)	Serum NO_3_^−^ ↑ Serum NO_2_^−^ ↑ MVIC peak torque ↔ Endurance ↑ HR ↔ RPE ↔ Lactate ↔ RMS EMG ↔

Note. Arrows indicate statistically significant differences as reported in the original studies (↑ increase, ↓ decrease); ↔ indicates no statistically significant difference.

**Table 10:** Chronic effects of beetroot juice supplementation on sprint and power performance.

Reference	Participants	Experimental Conditions	Supplementation Protocol	Supplement Source	Variables	Results
Hemmatinafar et al. [123]	W, semi-professional volleyball players (*n* = 12)	EC1: BRJ EC2: PLA	EC1: BRJ 400 mL/day (4.1 mmol) (120 min–38 h after)/2 days	Beetroot juice (red beet, Zarghan Lepoi Farms, Shiraz, Iran)	Wall-sit endurance, V-Sit reach flexibility test, Vertical jump height, PPT, Thigh swelling test	Wall-sit endurance ↑ V-Sit reach flexibility ↑ Vertical jump height ↔ PPT ↑ Thigh swelling ↓
Jonvik et al. [124]	M, recreational cyclists (*n* = 20), national talent speed-skaters (*n* = 22), Olympic track cyclists (*n* =10) (*n* = 52)	EC1: BRJ EC2: PLA	EC1: BRJ 140 mL/day (12.9 mmol) (morning)/6 days EC2: BRJ 140 mL/day (0.008 mmol) (morning)/6 days	Beet it (James White Drinks Ltd., Ipswich, UK)	3 × 30 s Wingate tests (cycle ergometer) (peak power, mean power, time to peak power, plasma NO_3_^−^, plasma NO_2_^−^)	Peak power ↔ Mean power ↔ Time to peak power ↓ Plasma NO_3_^−^ ↑ Plasma NO_2_^−^ ↑
Nyakayiru et al. [125]	M, trained amateur league soccer players (*n* = 32)	EC1: BRJ EC2: PLA	EC1: BRJ 140 mL/day (12.9 mmol) (evening)/6 days EC2: BRJ 140 mL/day (0 mmol) (evening)/6 days	Beet It (James White Drinks Ltd., Ipswich, UK)	Yo-Yo IR1 (total distance, peak HR, mean HR, post-test blood lactate, RPE, plasma NO_3_^−^/NO_2_^−^, saliva NO_3_^−^/NO_2_^−^)	Total distance ↑ Peak HR ↔ Mean HR ↔ Blood lactate ↔ RPE ↔ Plasma & saliva nitrate/nitrite ↑
Thompson et al. [126]	M (*n* = 18) and W (*n* = 12) Recreationally active adults (*n* = 30)	EC1: SIT EC2: SIT + BRJ EC3: SIT + potassium NO_3_^−^; KNO_3_	EC2: BRJ 2 × 70 mL/day (12.8 mmol) (morning, evening)/4 weeks	Beet it, James White Drinks, Ipswich, UK	4-week SIT: 3 sessions/week × 4 weeks (VO_2_peak, time-to-task failure, muscle lactate (3 min), plasma NO_3_^−^ response during severe exercise, phosphocreatine recovery time constant)	(EC2) VO_2_peak ↑ Time-to-task failure ↑ Muscle lactate↓ Plasma NO_3_^−^ fall during severe exercise ↓ PCr recovery time constant ↔
Yang et al. [127]	M, college athletes (*n* = 21)	EC1: BFR EC2: BFR + BRJ	EC2: BRJ 80 mL/day (8 mmol) (a set time each day)/4 weeks	Beetroot juice (M-ACTION, Shanghai, China)	Isokinetic BFR knee-extensor training: 5 sets (1 × 30 + 4 × 15 reps), 30% peak torque, 120°/s angular velocity, 40% limb occlusion (peak torque, peak power, average power, fatigue index, torque/power decline rate, 30 s anaerobic power)	Peak torque ↑ Peak power ↑ Average power ↑ Fatigue index ↓ Torque/power decline rate ↓ 30 s anaerobic power ↑

Note. Arrows indicate statistically significant differences as reported in the original studies (↑ increase, ↓ decrease); ↔ indicates no statistically significant difference.

**Table 11:** Effects of beetroot juice supplementation on team-sport performance and recovery.

Reference	Participants	Experimental Conditions	Supplementation Protocol	Supplement Source	Variables	Results
Martin et al. [114]	M (*n* = 9) and W (*n* = 7) moderately trained team-sport athletes (*n* = 16)	EC1: BRJ EC2: PLA	EC1: BRJ 70 mL/day (4.8 mmol) (120 min before)/1 day EC2: PLA 70 mL/day (120 min before)/1 day	Beet It (James White Drinks Ltd., Ipswich, UK)	Repeated 8 s maximal sprints, 30 s active recovery, individualized workload (200% peak ramp test), to exhaustion (Completed sprints, total work, mean/peak power, VO_2_, HR, lactate, RPE)	Sprint completed ↓ Total work ↓ Mean power ↔ Peak power ↔ HR ↔ RPE ↔
Clifford et al. [112]	M, team-sport players, soccer (*n* = 10), rugby (*n* = 5), basketball (*n* = 2) hockey (*n* = 2), handball (*n* = 1), (*n* = 20)	EC1: BRJ EC2: PLA	EC1: BRJ 2 × 250 mL/day (6.2 mmol) (morning, evening)/3 days	Love Beets Super Tasty Beetroot Juice (Gs Fresh Ltd., Cambridgeshire, UK)	Repeated Sprint Test, 30 s rest, forced deceleration zone 10 m, performed twice (CMJ height, Reactive Strength Index, PPT, sprint time, fatigue index)	CMJ height ↑ Reactive Strength Index ↑ PPT↑ Sprint time ↔ Fatigue index ↔
Thompson et al. [113]	M, recreational team-sport players (local field hockey, football and rugby) (*n* = 16)	EC1: BRJ EC2: PLA	EC1: BRJ 2 × 70 mL/day (6.4 mmol) (morning, evening)/7 days EC2: BRJ 2 × 70 mL/day (0.04 mmol) (morning, evening)/7 days	Beet it, James White Drinks Ltd., Ipswich, UK	Intermittent sprint test (peak power, mean power, fatigue index, VO, HR, RPE), choice reaction test, stroop, RVIP	Peak power ↑ Mean power ↑ Total work ↑ Fatigue index ↔ VO_2_ ↓ HR/RPE ↔ Choice reaction time ↑ Stroop ↑ Stroop ↔ RVIP↑
Thompson et al. [13]	M, team-sport players (local football, rugby and hockey teams) (*n* = 36)	EC1: BRJ EC2: PLA	EC1: BRJ 70 mL/day (6.4 mmol) (150 min before)/5 days EC2: BRJ 70 mL/day (0.04 mmol) (150 min before)/5 days	Beet it (James White Drinks Ltd., Ipswich, UK)	5 × 20 m maximal sprints (30 s walk recovery) + Yo-Yo IR1 (2 × 20 m shuttles, 10 s active recovery; to exhaustion) (5/10/20 m split times, reaction time, Stroop test, Yo-Yo IR1 distance, lactate, HR, RPE)	5 m split ↔ 10 m split ↑ 20 m split ↑ Reaction time ↑ Stroop performance ↑ Yo-Yo IR1 distance ↑ Lactate/HR/RPE ↔
Wylie et al. [38]	M, team-sport players (*n* = 10)	EC1: BRJ EC2: PLA	EC1: BRJ 2 × 70 mL/day (4.1 mmol) (morning, evening)/5 days EC2: BRJ 2 × 70 mL/day (0.04 mmol) (morning, evening)/5 days	Beet It (James White Drinks Ltd., Ipswich, UK)	24 × 6 s all-out sprints (24 s rest) + 7 × 30 s all-out (240 s rest) + 6 × 60 s self-paced (60 s rest) (mean power, total work, fatigue index, pulmonary gas exchange, blood lactate, plasma NO_2_^−^)	plasma NO_2_^−^ ↑ Mean power ↑ 7 × 30 s protocol ↔ 6 × 60 s protocol ↔ VO_2_ ↓ Blood lactate ↔

Note. Arrows indicate statistically significant differences as reported in the original studies (↑ increase, ↓ decrease); ↔ indicates no statistically significant difference.

## 6. Conclusions and Future Directions

Beetroot juice exerts its most consistent physiological actions through marked enhancement of nitrate–nitrite–NO bioavailability, improvements in redox balance, and microbiome-related modulation of nitrate-reduction capacity. Acute ingestion reliably increases circulating NOx and triggers short-term biochemical responses, yet these changes translate only minimally into classical metabolic markers such as glucose, insulin, lipids, or endocrine hormones. Chronic supplementation produces more sustained adaptations—including elevated fasting nitrate, enhanced oral and gut microbial nitrate reduction, and reductions in oxidative stress—although long-term effects on glycemic or lipid regulation remain inconsistent across studies. With respect to exercise performance, BRJ appears to benefit submaximal endurance tasks that depend on oxygen efficiency, whereas findings in high-intensity intermittent or strength–power exercise are variable and task-specific. Taken together, current evidence suggests that BRJ influences metabolic health primarily through NO-mediated and antioxidant pathways rather than through consistent modulation of conventional metabolic indices or uniform improvements in performance. This review should be interpreted in light of its narrative design, which does not aim to provide a systematic or exhaustive synthesis of the literature. Variations in the depth of evidence across domains reflect the current availability and maturity of human studies, underscoring the need for further research in less-studied areas. Future research should aim to clarify the sources of variability in metabolic responses to BRJ, including age-related declines in nitrate responsiveness, individual differences in oral and gut nitrate-reducing microbiota, and baseline cardiometabolic status. Multi-omics approaches integrating metabolomics, metagenomics, and mitochondrial bioenergetics are needed to delineate the mechanistic links between BRJ’s bioactive components and downstream metabolic outcomes beyond NO production alone. In the exercise domain, task-specific and responder-focused study designs are warranted to identify which individuals and exercise modalities benefit most from BRJ, particularly in high-intensity or neuromuscular contexts where current findings are inconsistent. Dose–response relationships, optimal timing strategies, chronic versus acute stacking effects, and interactions with diet, oral hygiene, and habitual nitrate intake also require systematic evaluation. Finally, translating BRJ research to clinical populations—including individuals with hypertension, endothelial dysfunction, insulin resistance, or age-related metabolic decline—remains an important avenue for determining its therapeutic potential in cardiometabolic health.

## Data Availability

No new data were created or analyzed in this study. Data sharing is not applicable to this article.

## Abbreviations

The following abbreviations are used in this manuscript:

1RM	One-repetition maximum
6MWT	Six-minute walk test
AD	Alzheimer’s disease
AFT	Afternoon
ATP	Adenosine triphosphate
AUClast	Area under the concentration-time curve to the last measurable concentration
AUG	Area under the glucose curve
baPWV	Brachial-ankle pulse wave velocity
BFR	Blood flow restriction
BP	Blood pressure
BRJ	Beetroot juice
Ca^2+^	Calcium ion
CAIx	Cerebral augmentation index
cBRS	Cardiovagal baroreflex sensitivity
CD11b	Cluster of differentiation 11b
cGMP	Cyclic guanosine monophosphate
CAF	Caffeine-supplemented group
CRP	C-reactive protein
CK	Creatine kinase
CMJ	Countermovement jump
CO	Cardiac output
CO_2_	Carbon dioxide
CON	Controlled
CR	Caloric restriction
CSA	Cross-section area
DBP	Diastolic blood pressure
DCA	Dynamic cerebral autoregulation
Deoxy-Hb	Deoxygenated hemoglobin
Deoxy-Mb	Deoxygenated myoglobin
DOMS	Delayed onset muscle soreness
EMG	Electromyography
EPM	Early postmenopausal
EVE	Evening
EXE	Exercise
FBF	Forearm blood-flow
FMD	Flow-mediated dilation
GLP-1	Glucagon-like peptide-1
GPS	Global positioning system
GSH	Reduced glutathione
GSSG	Oxidized glutathione
GTP	Guanosine triphosphate
Hb	Hemoglobin
HD	Hemodialysis
HF	High-frequency
HFpEF	Heart failure and preserved ejection fraction
HHb	Deoxyhemoglobin
HOMA-IR	Homeostatic model assessment-insulin resistance
HR	Heart rate
HRV	Heart rate variability
IHS	Isometric handgrip strength
IMTP	Isometric mid-thigh pull
IL-6	Interleukin-6
IPAQ	International physical activity questionnaire
LBNP	Lower-body negative pressure
LF	Low frequency
LPM	Late postmenopausal
M	Male participants
MAP	Mean arterial pressure
MIVC	Maximal isometric voluntary contraction
MORN	Morning
MPT	Mitochondrial permeability transition
MUFR	Motor unit firing rate
MUP	Motor unit potential
MVC	Maximal voluntary contraction
MVIC	Maximal voluntary isometric contraction
NF jiggle	Neuromuscular firing instability
NIRS	Near-infrared spectroscopy
NIT	Nitrate-supplemented group
NO	Nitric oxide
NOS	Nitric oxide synthase
NO_2_-	Nitrite
NO_3_-	Nitrate
NOx	Nitric oxide metabolites
NR	Not reported
O_2_	Oxygen
OGTT	Oral glucose tolerance test
OLD	Older group
oxLDL	Oxidized low-density lipoprotein
PCr	Phosphocreatine
PETCO_2_	Partial pressure of end-tidal carbon dioxide
PFC	Prefrontal Cortex
pH	Power of Hydrogen
PIx	Pulsatility indices
PKG	Protein kinase G
PLA	Placebo
PPO	Peak power output
PPT	Pain pressure threshold
PSTT	Progressive specific taekwondo test
PWV	Pulse wave variable
qNIRS	Quantitative near-infrared spectroscopy
Q_tw,pot_	Potentiated quadriceps twitch force
QUICKI	Quantitative insulin sensitivity check index
RBC	Red blood cell
REE	Resting energy expenditure
RER	Respiratory exchange ratio
RFD	Rate of force development
RIx	Resistivity indices
RMS	Root Mean Square
RoR	Retinoic acid-related orphan receptor
ROS	Reactive oxygen species
RPE	Rating of perceived exertion
RTD	Rate of torque development
RVIP	Rapid visual information processing
RyR	Ryanodine receptor
SBP	Systolic blood pressure
SCFAs	Short-chain fatty acids
SERCA	Sarco/endoplasmic reticulum Ca^2+^-ATPase
sGC	Soluble guanylate cyclase
SIT	Sprint interval training
SJ	Squat jump
SJFT	Sargent jump fatigue test
SpO_2_	Peripheral oxygen saturation
StO_2_	Tissue oxygen saturation
SVR	Systemic vascular resistance
T2D	Type 2 diabetes
TPR	Total peripheral resistance
TQR	Total quality recovery
TT	Time-trial
TTE	Time to exhaustion
VE	Minute ventilation
VEG	Vegetable-supplemented group
VE/VCO_2_	Ventilatory equivalent for carbon dioxide
VE/VO_2_	Ventilatory equivalent for oxygen
VLF	Very-low-frequency
VO_2_	Oxygen consumption
VO_2_peak	Peak oxygen consumption
W	Female participant
XOR	Xanthine oxidoreductase
YYIR1	Yo-Yo Intermittent Recovery Test Level 1
YN	Younger group

## References

[1] Jones A.M. (2014). Dietary nitrate supplementation and exercise performance. Sports Med..

[2] Lundberg J.O., Weitzberg E., Gladwin M.T. (2008). The nitrate-nitrite-nitric oxide pathway in physiology and therapeutics. Nat. Rev. Drug Discov..

[3] Stamler J.S., Meissner G. (2001). Physiology of nitric oxide in skeletal muscle. Physiol. Rev..

[4] Zamani H., De Joode M.E.J.R., Hossein I.J., Henckens N.F.T., Guggeis M.A., Berends J.E., De Kok T.M.C.M., Van Breda S.G.J. (2021). The benefits and risks of beetroot juice consumption: A systematic review. Crit. Rev. Food Sci. Nutr..

[5] Hadipour E., Taleghani A., Tayarani-Najaran N., Tayarani-Najaran Z. (2020). Biological effects of red beetroot and betalains: A review. Phytother. Res..

[6] Lundberg J.O., Carlstrom M., Weitzberg E. (2018). Metabolic Effects of Dietary Nitrate in Health and Di sease. Cell Metab..

[7] Webb A.J., Patel N., Loukogeorgakis S., Okorie M., Aboud Z., Misra S., Rashid R., Miall P., Deanfield J., Benjamin N. (2008). Acute blood pressure lowering, vasoprotective, and antiplatelet properties of dietary nitrate via bioconversion to nitrite. Hypertension.

[8] Kapil V., Khambata R.S., Robertson A., Caulfield M.J., Ahluwalia A. (2015). Dietary nitrate provides sustained blood pressure lowering in hypertensive patients: A randomized, phase 2, double-blind, placebo-controlled study. Hypertension.

[9] Larsen F.J., Schiffer T.A., Borniquel S., Sahlin K., Ekblom B., Lundberg J.O., Weitzberg E. (2011). Dietary inorganic nitrate improves mitochondrial efficiency in humans. Cell Metab..

[10] Hernandez A., Schiffer T.A., Ivarsson N., Cheng A.J., Bruton J.D., Lundberg J.O., Weitzberg E., Westerblad H. (2012). Dietary nitrate increases tetanic [Ca2+]i and contractile force in mouse fast-twitch muscle. J. Physiol..

[11] Wightman E.L., Haskell-Ramsay C.F., Thompson K.G., Blackwell J.R., Winyard P.G., Forster J., Jones A.M., Kennedy D.O. (2015). Dietary nitrate modulates cerebral blood flow parameters and cognitive performance in humans: A double-blind, placebo-controlled, crossover investigation. Physiol. Behav..

[12] Wylie L.J., Mohr M., Krustrup P., Jackman S.R., Ermiotadis G., Kelly J., Black M.I., Bailey S.J., Vanhatalo A., Jones A.M. (2013). Dietary nitrate supplementation improves team sport-specific intense intermittent exercise performance. Eur. J. Appl. Physiol..

[13] Thompson C., Vanhatalo A., Jell H., Fulford J., Carter J., Nyman L., Bailey S.J., Jones A.M. (2016). Dietary nitrate supplementation improves sprint and high-intensity intermittent running performance. Nitric Oxide.

[14] Lansley K.E., Winyard P.G., Bailey S.J., Vanhatalo A., Wilkerson D.P., Blackwell J.R., Gilchrist M., Benjamin N., Jones A.M. (2011). Acute dietary nitrate supplementation improves cycling time trial performance. Med. Sci. Sports Exerc..

[15] Govoni M., Jansson E.Å., Weitzberg E., Lundberg J.O. (2008). The increase in plasma nitrite after a dietary nitrate load is markedly attenuated by an antibacterial mouthwash. Nitric Oxide.

[16] Clements W.T., Lee S.R., Bloomer R.J. (2014). Nitrate ingestion: A review of the health and physical performance effects. Nutrients.

[17] Forstermann U., Sessa W.C. (2012). Nitric oxide synthases: Regulation and function. Eur. Heart J..

[18] Lidder S., Webb A.J. (2013). Vascular effects of dietary nitrate (as found in green leafy vegetables and beetroot) via the nitrate-nitrite-nitric oxide pathway. Br. J. Clin. Pharmacol..

[19] Hezel M.P., Weitzberg E. (2015). The oral microbiome and nitric oxide homoeostasis. Oral Dis..

[20] Modin A., Bjorne H., Herulf M., Alving K., Weitzberg E., Lundberg J.O. (2001). Nitrite-derived nitric oxide: A possible mediator of ‘acidic-metabolic’ vasodilation. Acta Physiol. Scand..

[21] Arnold W.P., Mittal C.K., Katsuki S., Murad F. (1977). Nitric oxide activates guanylate cyclase and increases guanosine 3′:5′-cyclic monophosphate levels in various tissue preparations. Proc. Natl. Acad. Sci. USA.

[22] Ignarro L.J., Buga G.M., Wood K.S., Byrns R.E., Chaudhuri G. (1987). Endothelium-derived relaxing factor produced and released from artery and vein is nitric oxide. Proc. Natl. Acad. Sci. USA.

[23] Ferguson S.K., Hirai D.M., Copp S.W., Holdsworth C.T., Allen J.D., Jones A.M., Musch T.I., Poole D.C. (2013). Impact of dietary nitrate supplementation via beetroot juice on exercising muscle vascular control in rats. J. Physiol..

[24] Casey D.P., Treichler D.P., Ganger C.T.t., Schneider A.C., Ueda K. (2015). Acute dietary nitrate supplementation enhances compensatory vasodilation during hypoxic exercise in older adults. J. Appl. Physiol..

[25] Velmurugan S., Kapil V., Ghosh S.M., Davies S., McKnight A., Aboud Z., Khambata R.S., Webb A.J., Poole A., Ahluwalia A. (2013). Antiplatelet effects of dietary nitrate in healthy volunteers: Involvement of cGMP and influence of sex. Free Radic. Biol. Med..

[26] Clifford T., Bell O., West D.J., Howatson G., Stevenson E.J. (2017). Antioxidant-rich beetroot juice does not adversely affect acute neuromuscular adaptation following eccentric exercise. J. Sports Sci..

[27] McNally B., Griffin J.L., Roberts L.D. (2016). Dietary inorganic nitrate: From villain to hero in metabolic disease?. Mol. Nutr. Food Res..

[28] Lee J.M., Johnson J.A. (2004). An important role of Nrf2-ARE pathway in the cellular defense mechanism. J. Biochem. Mol. Biol..

[29] Ma Q. (2013). Role of nrf2 in oxidative stress and toxicity. Annu. Rev. Pharmacol. Toxicol..

[30] Vidal P.J., Lopez-Nicolas J.M., Gandia-Herrero F., Garcia-Carmona F. (2014). Inactivation of lipoxygenase and cyclooxygenase by natural betalains and semi-synthetic analogues. Food Chem..

[31] Vulic J.J., Cebovic T.N., Canadanovic V.M., Cetkovic G.S., Djilas S.M., Canadanovic-Brunet J.M., Velicanski A.S., Cvetkovic D.D., Tumbas V.T. (2013). Antiradical, antimicrobial and cytotoxic activities of commercial beetroot pomace. Food Funct..

[32] Raish M., Ahmad A., Ansari M.A., Alkharfy K.M., Ahad A., Khan A., Ali N., Ganaie M.A., Hamidaddin M.A.A. (2019). Beetroot juice alleviates isoproterenol-induced myocardial damage by reducing oxidative stress, inflammation, and apoptosis in rats. 3 Biotech.

[33] Detopoulou P., Panagiotakos D.B., Antonopoulou S., Pitsavos C., Stefanadis C. (2008). Dietary choline and betaine intakes in relation to concentrations of inflammatory markers in healthy adults: The ATTICA study. Am. J. Clin. Nutr..

[34] Bailey S.J., Wilkerson D.P., Dimenna F.J., Jones A.M. (2009). Influence of repeated sprint training on pulmonary O_2_ uptake and muscle deoxygenation kinetics in humans. J. Appl. Physiol..

[35] Wylie L.J., Kelly J., Bailey S.J., Blackwell J.R., Skiba P.F., Winyard P.G., Jeukendrup A.E., Vanhatalo A., Jones A.M. (2013). Beetroot juice and exercise: Pharmacodynamic and dose-response relationships. J. Appl. Physiol..

[36] Bailey S.J., Winyard P., Vanhatalo A., Blackwell J.R., Dimenna F.J., Wilkerson D.P., Tarr J., Benjamin N., Jones A.M. (2009). Dietary nitrate supplementation reduces the O_2_ cost of low-intensity exercise and enhances tolerance to high-intensity exercise in humans. J. Appl. Physiol..

[37] Vanhatalo A., Bailey S.J., Blackwell J.R., DiMenna F.J., Pavey T.G., Wilkerson D.P., Benjamin N., Winyard P.G., Jones A.M. (2010). Acute and chronic effects of dietary nitrate supplementation on blood pressure and the physiological responses to moderate-intensity and incremental exercise. Am. J. Physiol.-Regul. Integr. Comp. Physiol..

[38] Wylie L.J., Bailey S.J., Kelly J., Blackwell J.R., Vanhatalo A., Jones A.M. (2016). Influence of beetroot juice supplementation on intermittent exercise performance. Eur. J. Appl. Physiol..

[39] Miller G.D., Collins S., Ives J., Williams A., Basu S., Kim-Shapiro D.B., Berry M.J. (2023). Efficacy and Variability in Plasma Nitrite Levels during Long-Term Supplementation with Nitrate Containing Beetroot Juice. J. Diet. Suppl..

[40] Wilkerson D.P., Hayward G.M., Bailey S.J., Vanhatalo A., Blackwell J.R., Jones A.M. (2012). Influence of acute dietary nitrate supplementation on 50 mile time trial performance in well-trained cyclists. Eur. J. Appl. Physiol..

[41] Garnacho-Castano M.V., Palau-Salva G., Serra-Paya N., Ruiz-Hermosel M., Berbell M., Vinals X., Bataller M.G., Carbonell T., Vilches-Saez S., Cobo E.P. (2020). Understanding the effects of beetroot juice intake on CrossFit performance by assessing hormonal, metabolic and mechanical response: A randomized, double-blind, crossover design. J. Int. Soc. Sports Nutr..

[42] Jurga J., Samborowska E., Zielinski J., Olek R.A. (2024). Effects of Acute Beetroot Juice and Sodium Nitrate on Selected Blood Metabolites and Response to Transient Ischemia: A Crossover Randomized Clinical Trial. J. Nutr..

[43] Heredia-Martinez A., Rosa-Diez G., Ferraris J.R., Sohlenius-Sternbeck A.K., Nihlen C., Olsson A., Lundberg J.O., Weitzberg E., Carlstrom M., Krmar R.T. (2022). Plasma Nitrate and Nitrite Kinetics after Single Intake of Beetroot Juice in Adult Patients on Chronic Hemodialysis and in Healthy Volunteers: A Randomized, Single-Blind, Placebo-Controlled, Crossover Study. Nutrients.

[44] Fuchs D., Nyakayiru J., Draijer R., Mulder T.P., Hopman M.T., Eijsvogels T.M., Thijssen D.H. (2016). Impact of flavonoid-rich black tea and beetroot juice on postprandial peripheral vascular resistance and glucose homeostasis in obese, insulin-resistant men: A randomized controlled trial. Nutr. Metab..

[45] Tyler A.P., Linder B.A., Ricart K., Behrens C.E., Ovalle F., Patel R.P., Fisher G. (2024). The Effects of Acute Beetroot Juice Intake on Glycemic and Blood Pressure Responses When Controlling for Medication in Individuals with Type 2 Diabetes: A Pilot Study. Nutrients.

[46] Shepherd A.I., Wilkerson D.P., Fulford J., Winyard P.G., Benjamin N., Shore A.C., Gilchrist M. (2016). Effect of nitrate supplementation on hepatic blood flow and glucose homeostasis: A double-blind, placebo-controlled, randomized control trial. Am. J. Physiol.-Gastrointest. Liver Physiol..

[47] Babateen A.M., Shannon O.M., O’Brien G.M., Okello E., Khan A.A., Rubele S., Wightman E., Smith E., McMahon N., Olgacer D. (2021). Acceptability and Feasibility of a 13-Week Pilot Randomised Controlled Trial Testing the Effects of Incremental Doses of Beetroot Juice in Overweight and Obese Older Adults. Nutrients.

[48] Alharbi M., Chiurazzi M., Nasti G., Muscariello E., Mastantuono T., Koechl C., Stephan B.C.M., Shannon O.M., Colantuoni A., Siervo M. (2023). Caloric Restriction (CR) Plus High-Nitrate Beetroot Juice Does Not Amplify CR-Induced Metabolic Adaptation and Improves Vascular and Cognitive Functions in Overweight Adults: A 14-Day Pilot Randomised Trial. Nutrients.

[49] Giampaoli O., Ieno C., Sciubba F., Spagnoli M., Miccheli A., Tomassini A., Aureli W., Fattorini L. (2023). Metabolic Biomarkers of Red Beetroot Juice Intake at Rest and after Physical Exercise. Nutrients.

[50] Fejes R., Seneca J., Pjevac P., Lutnik M., Weisshaar S., Pilat N., Steiner R., Wagner K.H., Woodman R.J., Bondonno C.P. (2025). Increased Nitrate Intake from Beetroot Juice Over 4 Weeks Changes the Composition of the Oral, But Not the Intestinal Microbiome. Mol. Nutr. Food Res..

[51] Vanhatalo A., L’Heureux J.E., Black M.I., Blackwell J.R., Aizawa K., Thompson C., Williams D.W., van der Giezen M., Winyard P.G., Jones A.M. (2025). Ageing modifies the oral microbiome, nitric oxide bioavailability and vascular responses to dietary nitrate supplementation. Free Radic. Biol. Med..

[52] Wang Y., Do T., Marshall L.J., Boesch C. (2023). Effect of two-week red beetroot juice consumption on modulation of gut microbiota in healthy human volunteers—A pilot study. Food Chem..

[53] Rowland S.N., James L.J., O’Donnell E., Bailey S.J. (2023). Influence of acute dietary nitrate supplementation timing on nitrate metabolism, central and peripheral blood pressure and exercise tolerance in young men. Eur. J. Appl. Physiol..

[54] Fejes R., Lutnik M., Weisshaar S., Pilat N., Wagner K.H., Stuger H.P., Peake J.M., Woodman R.J., Croft K.D., Bondonno C.P. (2024). Increased nitrate intake from beetroot juice over 4 weeks affects nitrate metabolism, but not vascular function or blood pressure in older adults with hypertension. Food Funct..

[55] Bonilla Ocampo D., Paipilla A., Marín E., Vargas-Molina S., Petro J., Pérez-Idárraga A. (2018). Dietary Nitrate from Beetroot Juice for Hypertension: A Systematic Review. Biomolecules.

[56] van der Avoort C.M.T., Jonvik K.L., Nyakayiru J., van Loon L.J.C., Hopman M.T.E., Verdijk L.B. (2020). A Nitrate-Rich Vegetable Intervention Elevates Plasma Nitrate and Nitrite Concentrations and Reduces Blood Pressure in Healthy Young Adults. J. Acad. Nutr. Diet..

[57] Volino-Souza M., de Oliveira G.V., Alvares T.S. (2018). A single dose of beetroot juice improves endothelial function but not tissue oxygenation in pregnant women: A randomised clinical trial. Br. J. Nutr..

[58] Richards J.C., Racine M.L., Hearon C.M., Kunkel M., Luckasen G.J., Larson D.G., Allen J.D., Dinenno F.A. (2018). Acute ingestion of dietary nitrate increases muscle blood flow via local vasodilation during handgrip exercise in young adults. Physiol. Rep..

[59] Curry B.H., Bond V., Pemminati S., Gorantla V.R., Volkova Y.A., Kadur K., Millis R.M. (2016). Effects of a Dietary Beetroot Juice Treatment on Systemic and Cerebral Haemodynamics- A Pilot Study. J. Clin. Diagn. Res..

[60] Worley M.L., Reed E.L., Chapman C.L., Kueck P., Seymour L., Fitts T., Zazulak H., Schlader Z.J., Johnson B.D. (2023). Acute beetroot juice consumption does not alter cerebral autoregulation or cardiovagal baroreflex sensitivity during lower-body negative pressure in healthy adults. Front. Hum. Neurosci..

[61] Chapman C.L., Schlader Z.J., Reed E.L., Worley M.L., Johnson B.D. (2021). Acute Beetroot Juice Ingestion Does Not Alter Renal Hemodynamics during Normoxia and Mild Hypercapnia in Healthy Young Adults. Nutrients.

[62] Bock J.M., Ueda K., Schneider A.C., Hughes W.E., Limberg J.K., Bryan N.S., Casey D.P. (2018). Inorganic nitrate supplementation attenuates peripheral chemoreflex sensitivity but does not improve cardiovagal baroreflex sensitivity in older adults. Am. J. Physiol. Heart Circ. Physiol..

[63] Siervo M., Lara J., Jajja A., Sutyarjoko A., Ashor A.W., Brandt K., Qadir O., Mathers J.C., Benjamin N., Winyard P.G. (2015). Ageing modifies the effects of beetroot juice supplementation on 24-hour blood pressure variability: An individual participant meta-analysis. Nitric Oxide.

[64] Bahadoran Z., Mirmiran P., Kabir A., Azizi F., Ghasemi A. (2017). The Nitrate-Independent Blood Pressure-Lowering Effect of Beetroot Juice: A Systematic Review and Meta-Analysis. Adv. Nutr..

[65] Jones T., Dunn E.L., Macdonald J.H., Kubis H.-P., McMahon N., Sandoo A. (2019). The effects of beetroot juice on blood pressure, microvascular function and large-vessel endothelial function: A randomized, double-blind, placebo-controlled pilot study in healthy older adults. Nutrients.

[66] Delgado Spicuzza J.M., Gosalia J., Zhong L., Bondonno C., Petersen K.S., De Souza M.J., Alipour E., Kim-Shapiro D.B., Somani Y.B., Proctor D.N. (2024). Seven-day dietary nitrate supplementation clinically significantly improves basal macrovascular function in postmenopausal women: A randomized, placebo-controlled, double-blind, crossover clinical trial. Front. Nutr..

[67] Pedrinolla A., Dorelli G., Porcelli S., Burleigh M., Mendo M., Martignon C., Fonte C., Dalle Carbonare L.G., Easton C., Muti E. (2025). Increasing nitric oxide availability via ingestion of nitrate-rich beetroot juice improves vascular responsiveness in individuals with Alzheimer’s Disease. Nitric Oxide.

[68] Alvares T.S., Pinheiro V., Proctor D.N., Junior C.A.C., Soares R.N. (2025). Twelve-week nitrate-rich beetroot extract supplementation improves lower limb vascular function and serum angiogenic potential in postmenopausal women. Am. J. Physiol.-Hear. Circ. Physiol..

[69] Osman M.M.A., Mullins E., Kleprlikova H., Wilkinson I.B., Lees C. (2023). Beetroot juice, exercise, and cardiovascular function in women planning to conceive. J. Hypertens..

[70] Stanaway L., Rutherfurd-Markwick K., Page R., Wong M., Jirangrat W., Teh K.H., Ali A. (2019). Acute Supplementation with Nitrate-Rich Beetroot Juice Causes a Greater Increase in Plasma Nitrite and Reduction in Blood Pressure of Older Compared to Younger Adults. Nutrients.

[71] Fejes R., Pilat N., Lutnik M., Weisshaar S., Weijler A.M., Kruger K., Draxler A., Bragagna L., Peake J.M., Woodman R.J. (2024). Effects of increased nitrate intake from beetroot juice on blood markers of oxidative stress and inflammation in older adults with hypertension. Free Radic. Biol. Med..

[72] Berlanga L.A., Lopez-Samanes A., Martin-Lopez J., de la Cruz R.M., Garces-Rimon M., Roberts J., Bertotti G. (2023). Dietary Nitrate Ingestion Does Not Improve Neuromuscular Performance in Male Sport Climbers. J. Hum. Kinet..

[73] López-Samanes Á., Pérez-López A., Moreno-Pérez V., Nakamura F.Y., Acebes-Sánchez J., Quintana-Milla I., Sánchez-Oliver A.J., Moreno-Pérez D., Fernández-Elías V.E., Domínguez R. (2020). Effects of Beetroot Juice Ingestion on Physical Performance in Highly Competitive Tennis Players. Nutrients.

[74] López-Samanes Á., Pérez-Lopez A., Morencos E., Muñoz A., Kühn A., Sánchez-Migallón V., Moreno-Pérez V., González-Frutos P., Bach-Faig A., Roberts J. (2023). Beetroot juice ingestion does not improve neuromuscular performance and match-play demands in elite female hockey players: A randomized, double-blind, placebo-controlled study. Eur. J. Nutr..

[75] Lopez-Samanes A., Ramos-Alvarez J.J., Miguel-Tobal F., Gaos S., Jodra P., Arranz-Munoz R., Dominguez R., Montoya J.J. (2022). Influence of Beetroot Juice Ingestion on Neuromuscular Performance on Semi-Professional Female Rugby Players: A Randomized, Double-Blind, Placebo-Controlled Study. Foods.

[76] Wei C., Vanhatalo A., Black M.I., Rajaram R., Massey G., Jones A.M. (2025). Dose-response relationship between dietary nitrate intake and nitric oxide congeners in various blood compartments and skeletal muscle: Differential effects on skeletal muscle torque and velocity. Free Radic. Biol. Med..

[77] Esen O., Bailey S.J., Stashuk D.W., Howatson G., Goodall S. (2024). Influence of nitrate supplementation on motor unit activity during recovery following a sustained ischemic contraction in recreationally active young males. Eur. J. Nutr..

[78] Esen O., Faisal A., Zambolin F., Bailey S.J., Callaghan M.J. (2022). Effect of nitrate supplementation on skeletal muscle motor unit activity during isometric blood flow restriction exercise. Eur. J. Appl. Physiol..

[79] Jones L., Bailey S.J., Rowland S.N., Alsharif N., Shannon O.M., Clifford T. (2022). The Effect of Nitrate-Rich Beetroot Juice on Markers of Exercise-Induced Muscle Damage: A Systematic Review and Meta-Analysis of Human Intervention Trials. J. Diet. Suppl..

[80] Munoz A., de la Rubia A., Lorenzo-Calvo J., Karayigit R., Garces-Rimon M., Lopez-Moreno M., Dominguez R., Scanlan A.T., Lopez-Samanes A. (2025). Multiday Beetroot Juice Ingestion Improves Some Aspects of Neuromuscular Performance in Semi-Professional, Male Handball Players: A Randomized, Double-Blind, Placebo-Controlled, Crossover Study. Int. J. Sport Nutr. Exerc. Metab..

[81] Tan R., Lincoln I.G., Paniagua K.K., Foster J.M., Wideen L.E., Gerardo R.T., Ornelas N.J., Tchaprazian I., Li J., Egiazarian M. (2025). The effect of dietary nitrate supplementation on resistance exercise performance: A dose–response investigation. Eur. J. Appl. Physiol..

[82] Benjamim C.J.R., da Silva L.S.L., Sousa Y.B.A., Rodrigues G.D.S., Pontes Y.M.M., Rebelo M.A., Goncalves L.D.S., Tavares S.S., Guimaraes C.S., da Silva Sobrinho A.C. (2024). Acute and short-term beetroot juice nitrate-rich ingestion enhances cardiovascular responses following aerobic exercise in postmenopausal women with arterial hypertension: A triple-blinded randomized controlled trial. Free Radic. Biol. Med..

[83] Engan H., Patrician A., Lodin-Sundstrom A., Johansson H., Melin M., Schagatay E. (2020). Spleen contraction and Hb elevation after dietary nitrate intake. J. Appl. Physiol..

[84] Hayes E., Alhulaefi S., Siervo M., Whyte E., Kimble R., Matu J., Griffiths A., Sim M., Burleigh M., Easton C. (2025). Inter-individual differences in the blood pressure lowering effects of dietary nitrate: A randomised double-blind placebo-controlled replicate crossover trial. Eur. J. Nutr..

[85] Horiuchi M., Rossetti G.M., Oliver S.J. (2022). Dietary nitrate supplementation effect on dynamic cerebral autoregulation in normoxia and acute hypoxia. J. Cereb. Blood Flow. Metab..

[86] Kelly J., Fulford J., Vanhatalo A., Blackwell J.R., French O., Bailey S.J., Gilchrist M., Winyard P.G., Jones A.M. (2013). Effects of short-term dietary nitrate supplementation on blood pressure, O_2_ uptake kinetics, and muscle and cognitive function in older adults. Am. J. Physiol. Regul. Integr. Comp. Physiol..

[87] Londono-Hoyos F., Zamani P., Beraun M., Vasim I., Segers P., Chirinos J.A. (2018). Effect of organic and inorganic nitrates on cerebrovascular pulsatile power transmission in patients with heart failure and preserved ejection fraction. Physiol. Meas..

[88] Raubenheimer K., Hickey D., Leveritt M., Fassett R., Ortiz de Zevallos Munoz J., Allen J.D., Briskey D., Parker T.J., Kerr G., Peake J.M. (2017). Acute Effects of Nitrate-Rich Beetroot Juice on Blood Pressure, Hemostasis and Vascular Inflammation Markers in Healthy Older Adults: A Randomized, Placebo-Controlled Crossover Study. Nutrients.

[89] Rogerson D., Aguilar Mora F.A., Young J.S., Klonizakis M. (2022). No effect of nitrate-rich beetroot juice on microvascular function and blood pressure in younger and older individuals: A randomised, placebo-controlled double-blind pilot study. Eur. J. Clin. Nutr..

[90] Babateen A.M., Shannon O.M., O’Brien G.M., Olgacer D., Koehl C., Fostier W., Mathers J.C., Siervo M. (2023). Moderate doses of dietary nitrate elicit greater effects on blood pressure and endothelial function than a high dose: A 13-week pilot study. Nutr. Metab. Cardiovasc. Dis..

[91] Lopez-Samanes A., Gomez Parra A., Moreno-Perez V., Courel-Ibanez J. (2020). Does Acute Beetroot Juice Supplementation Improve Neuromuscular Performance and Match Activity in Young Basketball Players? A Randomized, Placebo-Controlled Study. Nutrients.

[92] Daab W., Zghal F., Nassis G.P., Rebai H., Moalla W., Bouzid M.A. (2024). Chronic beetroot juice supplementation attenuates neuromuscular fatigue etiology during simulated soccer match play. Appl. Physiol. Nutr. Metab..

[93] Miraftabi H., Avazpoor Z., Berjisian E., Sarshin A., Rezaei S., Dominguez R., Reale R., Franchini E., Samanipour M.H., Koozehchian M.S. (2021). Effects of Beetroot Juice Supplementation on Cognitive Function, Aerobic and Anaerobic Performances of Trained Male Taekwondo Athletes: A Pilot Study. Int. J. Environ. Res. Public Health.

[94] Thompson K.G., Turner L., Prichard J., Dodd F., Kennedy D.O., Haskell C., Blackwell J.R., Jones A.M. (2014). Influence of dietary nitrate supplementation on physiological and cognitive responses to incremental cycle exercise. Respir. Physiol. Neurobiol..

[95] Babateen A.M., Shannon O.M., O’Brien G.M., Okello E., Smith E., Olgacer D., Koehl C., Fostier W., Wightman E., Kennedy D. (2022). Incremental Doses of Nitrate-Rich Beetroot Juice Do Not Modify Cognitive Function and Cerebral Blood Flow in Overweight and Obese Older Adults: A 13-Week Pilot Randomised Clinical Trial. Nutrients.

[96] Gao C., Gupta S., Adli T., Hou W., Coolsaet R., Hayes A., Kim K., Pandey A., Gordon J., Chahil G. (2022). The effects of dietary nitrate supplementation on endurance exercise performance and cardiorespiratory measures in healthy adults: A systematic review and meta-analysis. J. Int. Soc. Sports Nutr..

[97] Garnacho-Castaño M.V., Pleguezuelos-Cobo E., Berbel M., Irurtia A., Carrasco-Marginet M., Castizo-Olier J., Veiga-Herreros P., Faundez-Zanuy M., Serra-Payá N. (2024). Effects of acute beetroot juice intake on performance, maximal oxygen uptake, and ventilatory efficiency in well-trained master rowers: A randomized, double-blinded crossover study. J. Int. Soc. Sports Nutr..

[98] Ahmadpour A., Fashi M., Hemmatinafar M. (2024). Consuming Beetroot Juice Improves Slalom Performance and Reduces Muscle Soreness in Alpine Skiers under Hypoxic Conditions. Curr. Dev. Nutr..

[99] Shannon O.M., Barlow M.J., Duckworth L., Williams E., Wort G., Woods D., Siervo M., O’Hara J.P. (2017). Dietary nitrate supplementation enhances short but not longer duration running time-trial performance. Eur. J. Appl. Physiol..

[100] Muggeridge D.J., Howe C., Spendiff O., Pedlar C., James P.E., Easton C. (2014). A single dose of beetroot juice enhances cycling performance in simulated altitude. Med. Sci. Sports Exerc..

[101] Lowings S., Shannon O.M., Deighton K., Matu J., Barlow M.J. (2017). Effect of dietary nitrate supplementation on swimming performance in trained swimmers. Int. J. Sport Nutr. Exerc. Metab..

[102] Tan R., Wylie L.J., Thompson C., Blackwell J.R., Bailey S.J., Vanhatalo A., Jones A.M. (2018). Beetroot juice ingestion during prolonged moderate-intensity exercise attenuates progressive rise in O_2_ uptake. J. Appl. Physiol..

[103] Rasica L., Porcelli S., Marzorati M., Salvadego D., Vezzoli A., Agosti F., De Col A., Tringali G., Jones A.M., Sartorio A. (2018). Ergogenic effects of beetroot juice supplementation during severe-intensity exercise in obese adolescents. Am. J. Physiol.-Regul. Integr. Comp. Physiol..

[104] Aucouturier J., Boissiere J., Pawlak-Chaouch M., Cuvelier G., Gamelin F.X. (2015). Effect of dietary nitrate supplementation on tolerance to supramaximal intensity intermittent exercise. Nitric Oxide.

[105] Neteca J., Veseta U., Liepina I., Volgemute K., Dzintare M., Babarykin D. (2024). Effect of Beetroot Juice Supplementation on Aerobic Capacity in Female Athletes: A Randomized Controlled Study. Nutrients.

[106] Porcelli S., Ramaglia M., Bellistri G., Pavei G., Pugliese L., Montorsi M., Rasica L., Marzorati M. (2015). Aerobic Fitness Affects the Exercise Performance Responses to Nitrate Supplementation. Med. Sci. Sports Exerc..

[107] Boorsma R.K., Whitfield J., Spriet L.L. (2014). Beetroot juice supplementation does not improve performance of elite 1500-m runners. Med. Sci. Sports Exerc..

[108] Cuenca E., Jodra P., Pérez-López A., González-Rodríguez L.G., Fernandes Da Silva S., Veiga-Herreros P., Domínguez R. (2018). Effects of Beetroot Juice Supplementation on Performance and Fatigue in a 30-s All-Out Sprint Exercise: A Randomized, Double-Blind Cross-Over Study. Nutrients.

[109] Rimer E.G., Peterson L.R., Coggan A.R., Martin J.C. (2016). Increase in Maximal Cycling Power with Acute Dietary Nitrate Supplementation. Int. J. Sports Physiol. Perform..

[110] Jurado-Castro J.M., Campos-Perez J., Ranchal-Sanchez A., Duran-Lopez N., Dominguez R. (2022). Acute Effects of Beetroot Juice Supplements on Lower-Body Strength in Female Athletes: Double-Blind Crossover Randomized Trial. Sports Health.

[111] Demirli A., Gokcelik E., Moghanlou A.E., Ocak M.H., Terzi M., Yamaner E., Atici M., Akyuz O., Toy A.B. (2025). Acute beetroot juice supplementation enhances judo-specific performance, explosive power, and muscular strength in recreational adolescent judokas: A randomized crossover trial. Front. Nutr..

[112] Clifford T., Berntzen B., Davison G.W., West D.J., Howatson G., Stevenson E.J. (2016). Effects of Beetroot Juice on Recovery of Muscle Function and Performance between Bouts of Repeated Sprint Exercise. Nutrients.

[113] Thompson C., Wylie L.J., Fulford J., Kelly J., Black M.I., McDonagh S.T.J., Jeukendrup A.E., Vanhatalo A., Jones A.M. (2015). Dietary nitrate improves sprint performance and cognitive function during prolonged intermittent exercise. Eur. J. Appl. Physiol..

[114] Martin K., Smee D., Thompson K.G., Rattray B. (2014). No improvement of repeated-sprint performance with dietary nitrate. Int. J. Sports Physiol. Perform..

[115] Moreno B., Morencos E., Vicente-Campos D., Munoz A., Gonzalez-Garcia J., Veiga S. (2022). Effects of beetroot juice intake on repeated performance of competitive swimmers. Front. Physiol..

[116] Moreno-Heredero B., Morencos E., Morais J., Barbosa T.M., Veiga S. (2024). A Single Dose of Beetroot Juice not Enhance Performance during Intervallic Swimming Efforts. J. Sports Sci. Med..

[117] Zhang J., Dai Z., Heung-Sang Wong S., Zheng C., Tsz-Chun Poon E. (2024). Acute effects of various doses of nitrate-rich beetroot juice on high-intensity interval exercise responses in women: A randomized, double-blinded, placebo-controlled, crossover trial. J. Int. Soc. Sports Nutr..

[118] Carriker C.R., Vaughan R.A., Vandusseldorp T.A., Johnson K.E., Beltz N.M., McCormick J.J., Cole N.H., Gibson A.L. (2016). Nitrate-Containing Beetroot Juice Reduces Oxygen Consumption During Submaximal Exercise in Low but Not High Aerobically Fit Male Runners. J. Exerc. Nutr. Biochem..

[119] Pinna M., Roberto S., Milia R., Marongiu E., Olla S., Loi A., Migliaccio G.M., Padulo J., Orlandi C., Tocco F. (2014). Effect of beetroot juice supplementation on aerobic response during swimming. Nutrients.

[120] Esen O., Karayigit R., Peart D.J. (2023). Acute beetroot juice supplementation did not enhance intermittent running performance in trained rugby players. Eur. J. Sport Sci..

[121] Montalvo-Alonso J.J., Del Val-Manzano M., Ferragut C., Valadés D., López-Samanes Á., Domínguez R., Pérez-López A. (2025). Single and combined effect of beetroot juice and caffeine intake on muscular strength, power and endurance performance in resistance-trained males. Sci. Rep..

[122] Wang L., Zhao R., Yan Y., Zhang H., Yan R., Zhu Y., Han Z., Qu Y., Wang R., Li Y. (2025). Effects of dietary nitrate supplementation on isometric performance and physiological responses in college bodybuilders: A randomized, double-blind, crossover study. Front. Nutr..

[123] Hemmatinafar M., Zaremoayedi L., Koushkie Jahromi M., Alvarez-Alvarado S., Wong A., Niknam A., Suzuki K., Imanian B., Bagheri R. (2023). Effect of Beetroot Juice Supplementation on Muscle Soreness and Performance Recovery after Exercise-Induced Muscle Damage in Female Volleyball Players. Nutrients.

[124] Jonvik K.L., Nyakayiru J., Van Dijk J.W., Maase K., Ballak S.B., Senden J.M.G., Van Loon L.J.C., Verdijk L.B. (2018). Repeated-sprint performance and plasma responses following beetroot juice supplementation do not differ between recreational, competitive and elite sprint athletes. Eur. J. Sport Sci..

[125] Nyakayiru J., Jonvik K., Trommelen J., Pinckaers P., Senden J., Van Loon L., Verdijk L. (2017). Beetroot Juice Supplementation Improves High-Intensity Intermittent Type Exercise Performance in Trained Soccer Players. Nutrients.

[126] Thompson C., Vanhatalo A., Kadach S., Wylie L.J., Fulford J., Ferguson S.K., Blackwell J.R., Bailey S.J., Jones A.M. (2018). Discrete physiological effects of beetroot juice and potassium nitrate supplementation following 4-wk sprint interval training. J. Appl. Physiol..

[127] Yang X., Lu Y., Xu H., Liu Q., Yun D.H., Moon Y.J., Quan H., Lee S.K. (2025). Synergistic effects of blood flow restriction training and beetroot juice supplementation on knee extensor strength and fatigue resistance in college athletes. Biol. Sport.

